# Fabrication and characterization of a scalable surface textured with pico-liter oil drops for mechanistic studies of bacteria-oil interactions

**DOI:** 10.1038/s41598-018-25812-y

**Published:** 2018-05-15

**Authors:** Maryam Jalali, Andrew R. White, James Marti, Jian Sheng

**Affiliations:** 1 Department of Engineering, Texas A&M University, Corpus Christi, Texas USA; 2 Nano Fabrication Center, University of Minnesota, Twin City, Minnesota USA

## Abstract

Texturing a large surface with oily micro-drops with controlled size, shape and volume provides an unprecedented capability in investigating complex interactions of bacteria, cells and interfaces. It has particular implications in understanding key microbial processes involved in remediation of environmental disasters, such as Deepwater Horizon oil spill. This work presents a development of scalable micro-transfer molding to functionalize a substrate with oily drop array to generate a microcosm mimicking bacteria encountering a rising droplet cloud. The volume of each drop within a large “printed” surface can be tuned by varying base geometry and area with characteristic scales from 5 to 50 ***μm***. Contrary to macroscopic counterparts, drops with non-Laplacian shapes, i.e. sharp corners, that appears to violate Young-Laplacian relationship locally, are produced. Although the drop relaxes into a spherical cap with constant mean curvature, the contact line with sharp corners remains pinned. Relaxation times from initial to asymptotic shape require extraordinarily long time (>7 days). We demonstrate that non-Laplacian drops are the direct results of self-pinning of contact line by nanoparticles in the oil. This technique has been applied to study biofilm formation at the oil-water interface and can be readily extended to other colloidal fluids.

## Introduction

Nanofabrication and microfluidics are playing an ever increasing role in studying chemical and biological processes^1^. This is particularly true in studying complex microbial processes such as aggregation and biofilm formation^2^, microbial streamer formation^3^, quorum sensing^4^, as well as particle mobility and cellular motility near interfaces^4,5^. In the context of oil spills, bacteria and other microorganisms have long been thought to play a significant role in effectively degrading various hydrocarbons — short-chain, long-chain and many aromatic compounds, including polycyclic aromatic hydrocarbons (PAH) — by either promoting interfacial instability at oil-water interface via biosurfactant^6^ to cause further breakdown or utilizing them as available carbon source in their metabolisms^7^. Since most of these hydrocarbons have low solubility in water (e.g. only ~7% by weight of the crude is soluble), in both degradation scenarios, these planktonic microbes must first make direct contact to the oil-water interface to access the large amount of insoluble hydrocarbon. The interactions between microbes and interfaces involve many complex processes including cell swimming and reorientation mechanisms, cell adsorption and desorption, cell motility modes, remodeling of interface, and impacts on the interface due to cell motions. To address these complex processes, they must be investigated at the comparative scales relevant to individual micro-organism and local interface topology they interact with, e.g. O(1–10 *μ*m). Furthermore to faithfully elucidate the dynamic processes involved in the microbial biodegradation of oil, the oil interface must be immobilized. To best of our knowledge, there is no experimental method that is capable of tracking individual bacteria and simultaneously observing their impact on interfaces. In this study, we present a technique that is capable of generating an immobilized oil-water interface with relevant length scale textures that enables studies of bacteria – interface interactions and assesses the key degradation mechanisms of oil by bacteria.

A number of techniques have been developed in the past decades to generate stationary microscale fluid interfaces. Inkjet printing (or 3D printing)^8–10^ can be used to deposit large volumes of different materials such as polymers, lubricants, UV curable resins, and biological solutions^8,11,12^ on a solid substrate, but the techniques lack sophisticated volume control and often require complex printing apparatuses which are difficult to be incorporated into microfluidic microcosm experimentations. Ismagilov and co-workers have developed “SlipChip” microfluidics to generate micro-drops with discreet volume control using the shearing motion of two plates engraved with wells and ducts^13,14^, allowing microliters of a solution to be evenly distributed among hundreds of micro-compartments^15,16^. However the technique must trap droplets between two parallel plates constantly and limits the exposed reactive surfaces. Mirkin and coworkers have developed Dip-Pen Nanolithography (DPN), capable of depositing material with the tip of an atomic force microscope (AFM) probe and later extended it to a multiple pen configuration^17–19^. This technique can pattern the substrate with a wide variety of materials from small organic molecules to biological polymers, and from colloidal particles to metallic material^19–21^. Although the technique can achieve easily nanometer resolutions, they are limited by the small amount of materials transferrable to the substrate and make structuring a larger surface a prohibitive task. Additional micro-drop generation methods employ surfaces with super-hydrophobic and super-hydrophilic regions to create micro-drops during a thin film drainage^22–25^. These techniques carry many advantages for aqueous solutions^26^, but their applications to amphiphilic materials like crude oil have not been reported in literature.

Whitesides and colleagues have pioneered another class of methods, namely soft lithography, which is a technique based on printing and molding using soft polymeric stamps containing desired features to fabricate microstructures^27–30^. Briefly, soft lithography refers to a wide range of techniques including Replica Molding (REM), Micro-Contact Printing (µCP), Micro-Molding in Capillaries (MIMIC), Micro-Transfer Molding (µTM), and Solvent-Assisted Micro-Molding (SAMIM)^30,31^, which deposit a wide variety of materials including biomolecules, polymers and nanomaterials^32^ with incredible precision and resolutions. Among these collective techniques, µCP^33–35^ has demonstrated the extraordinary capability in transferring monolayer (nm) and two-dimensional (um) features of semiconductors, metal and metal-oxide materials to substrates and subsequently in forming three-dimensional micro-structures by multiple consecutive printing. Later on, the method has been applied to patterning substrates with monolayer biomolecular materials^36^. Although widely used, it has not been applied to colloidal suspensions to form surface mounted micro-drops. MIMIC and SAMIM have been also developed to micro-pattern the substrate with photo-/thermal-curable polymers, which can achieve large scale patterning with micron and sub-micron resolution^37,38^. While MIMIC requires the printing materials to be cured before the mold can be released, SAMIM, an extension of MIMIC, absorbs the solvent by the mold instead^39,40^. However, these techniques have not been known to deposit large volume (>picoliter) of liquid to form a micro-drop array. To transfer a large volume of liquid and form micro-scale 3D structures, µTM^41^ has also been developed. However, in literatures it has only been successfully applied to fabricating three-dimensional complex structures with thermally or photochemically curable materials^40,42–44^, not oily fluids.

In this paper, we present a simple but robust approach to print arrays of isolated stable pico-liter oil drops with well-defined geometry and volume using micro-transfer molding. To the best of our knowledge, µTM has not been employed to generate surface mounted oily colloidal drops of 4–5 µm in height and pico-liters in volume without solidification. We will demonstrate that µTM can be extended to oily fluids to generate thousands of surface mounted oily drops with a simple one-step stamping procedure. We will further show that owing to the presence of amphiphilic nanoparticles, this simple fabrication technique also provides us with exquisite control over drops’ shape and volume as well as their 3D profiles. Results on printed surfaces with different drop size and base shapes are first shown in Results and Discussion. It is followed by detailed characterization of the shape of printed oil droplets and their temporal evolution. Noting that the proposed method allows us to print drops with square bases, we offer a plausible explanation based on the self-pinning mechanism of the drop contact line induced by adsorbed nanoparticles as well as a series of verification experiments, which is followed by the wettability of textured surface. In Section: Materials and methods, we discuss the µTM technique in great details.

## Results and Discussion

### Substrates with micro oil drops

**Figure 1:**
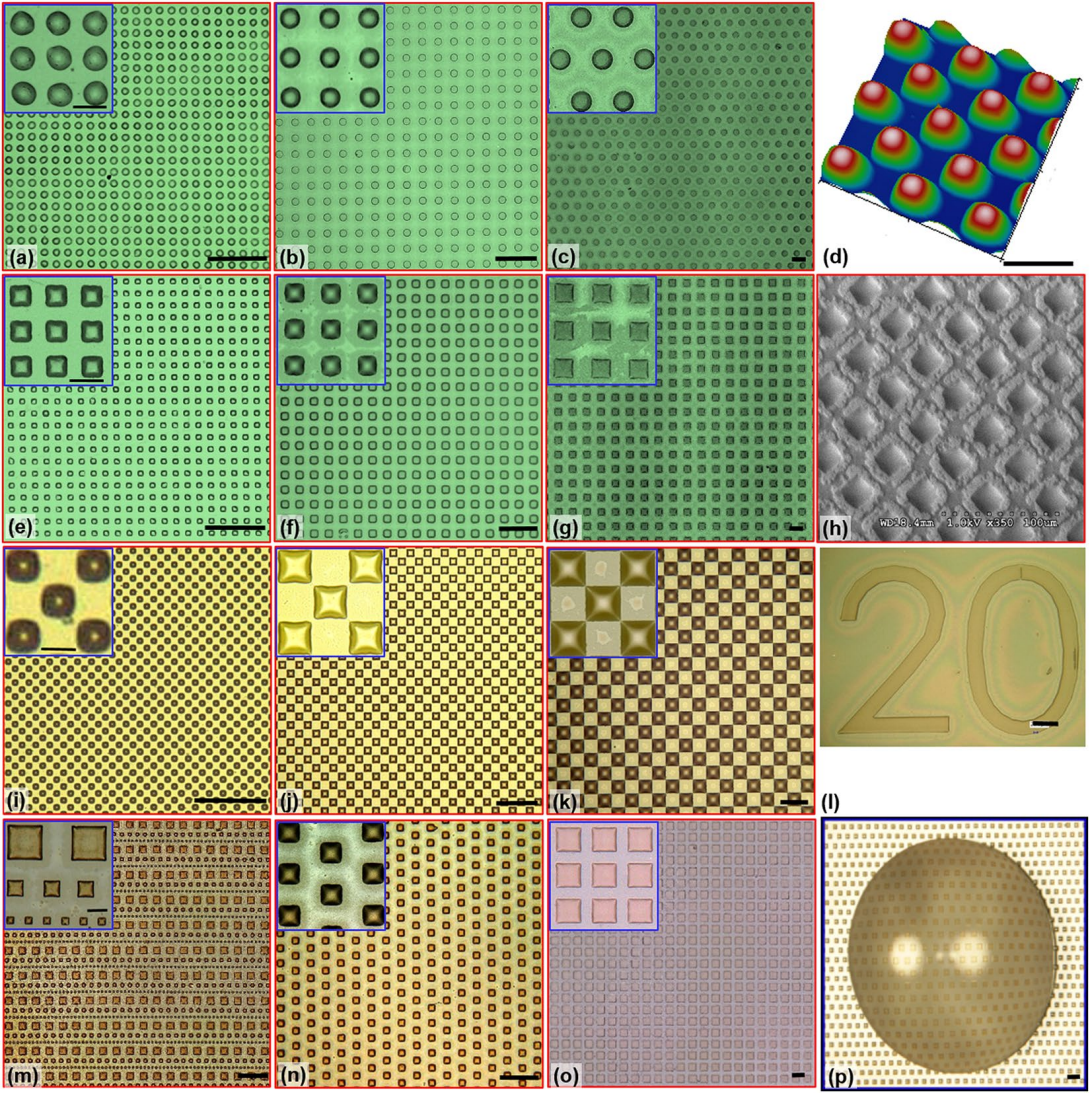
Sample surfaces textured with pico-liter drops. Drops with circular contact line of diameter of (**a**) 10, (**b**) 20, and (**c**) 50 *μm*. Drops with square base of (**e**) 10 × 10, (**f**) 20 × 20, and (**g**) 50 × 50 *μm*. Drops with square base in the pattern of a checker board: (**i**) 10 × 10, (**j**) 20 × 20, and (**k**) 50 × 50 *μm*. Drops with square base of (**m**) mixed sizes of 10, 20, and 50 *μm*, (**n**) 20 × 20 *μm* in a staggered pattern, and (**o**) 70 × 70 *μm* in regular pattern. Insets: blowouts of an array of 3 × 3 corresponding drops. (**d**) AFM measurement of the print of drops with the square of 50 × 50 *μm*. (**h**) SEM micrograph of a print of drops with the square of 20 × 20 *μm* imaged at 2.5 kV. (**l**) oil drops with a base of the letter 20. (**p**) micrograph of a water drop situated right above the substrate textured with pico-liter drops with square base. The oil drops are stable and remain attached to the glass substrate. Scale: 50 *μm*.

Micro transfer molding is successfully applied to print crude oil drops onto a tricholor(octadecyl)silane (OTS)-treated glass. It is demonstrated that *μ*TM can print oil drops with features of different base shapes and sizes as well as texture the surface with complicated spatial configurations (Table 1). Micrographs of sample prints superimposed with the corresponding magnified images (Inset at top right corner of each panel that shows drop details in Fig. 1) demonstrate that the technique is capable of generating precise individual micro-scale oil drops over a large area (3 × 3 *cm*^2^) with more than 90% usable area of the total printed surface. Note that the print size is only limited by stamps. Figure 1a–c show micrographs of oil drops with circular base but of various diameters (e.g.10, 20, and 50 *μm* corresponding to Column 1, 2, and 3 respectively). The well-organized arrays of drops are clearly formed over a large area. The insets show slight deviations from a circular base. The severity of this deviation decreases with the increase of drop diameter, e.g. 10 *μm* circular-base drop (CIR10) has more irregular circular base than 50 *μm* drop (CIR50). This trend is expected since the contact line of a smaller drop is more strongly affected by the surface inhomogeneity than those of the larger drops. Besides the conventional circular-base drops, the technique is also capable of printing drops with a non-traditional base containing sharp corners (square base drops shown in Fig. 1e–g). Uniformity of oily textures at corresponding scales are clearly observed. The sharp corners are “unexpectedly” produced (Insets in Fig. 1e–g), while sharpness increases with drop size. To further demonstrate the robustness of the technique, a checkerboard texture is also printed (Fig. 1i–k), within which each individual drop maintains its base shape even when the corners of each drop are in close contact with those of neighboring ones (Fig. 1k). In addition to those regular textures, the technique is capable of texturing the surface with oily drops with stamps of any designs, e.g. mixed square drops (Fig. 1m), staggered 20 *μm* square drops (Fig. 1n), larger square drops (Fig. 1o) and even letters (Fig. 1l).

**Table 1:** Characteristic dimension and geometry of features.

	Square				Circle		
	Length *d*_*f*_ (*μm*)	Pitch *w* (*μm*)	Height *h* (*μm*)		Diameter *d*_*f*_ (*μm*)	Pitch *w* (*μm*)	Height *h* (*μm*)
SQ10	10	20	6	CIR10	10	20	6
SQ20	20	40	6	CIR20	20	40	6
SQ50	50	100	6	CIR50	50	100	6
Configuration							
Regular	Staggered		Checkerboard	Regular		Staggered

To our expectation, these printed oil drops are surprisingly robust to meet our objectives: experimentations of biofilm growth over these surfaces. It is evident in Fig. 1d and h that these oil drops survive the rigorous environment of AFM and SEM. Additionally, the oily textured substrate is very stable when submerged in various aqueous environments for days which biofilm experimentation demands. Anecdotally, as a water drop is placed over the printed oily substrate (Fig. 1p), no detachment of drops is observed and texture maintains its integrity. Note that the water drop acts as a magnifying lens showing the pico-liter oil drops underneath it. These unexpected robust characteristics of these printed oil drops lead to the following questions:

(i) What is the shape of a pico-liter drop;
(ii) What is the mechanism allowing the drop to maintain its shape; and
(iii) Will this technique apply to other materials to texture a surface?

### Asymptotic shape of a pico-liter drop with a pinned contact line

In the absence of inertia, the shape of a pico-liter drop is expected to be determined by interfacial forces and fluids viscosity, among which interfacial forces determine the shape of a drop and viscosity affects the temporal scale of these changes. Six types of drops (CIR10, CIR20, CIR50, SQ10, SQ20 and SQ50) are printed using fresh crude to first address the asymptotic shape of a pico-liter drop with pinned contact lines. These AFM measurements are performed one hour immediately after the printing. Our time resolved study has shown that for fresh crude, the drop will reach its equilibrium shape within one hour. Due to the cost, we only randomly select three drops over each print for AFM measurements based on the initial microscopic inspection. Although the AFM measurement does not provide statistical robust observation quantitatively, the initial visual inspection ensures that each drop measured represents the major population of drops in the print.

### Shape of drop with a circular contact line

**Figure 2:**
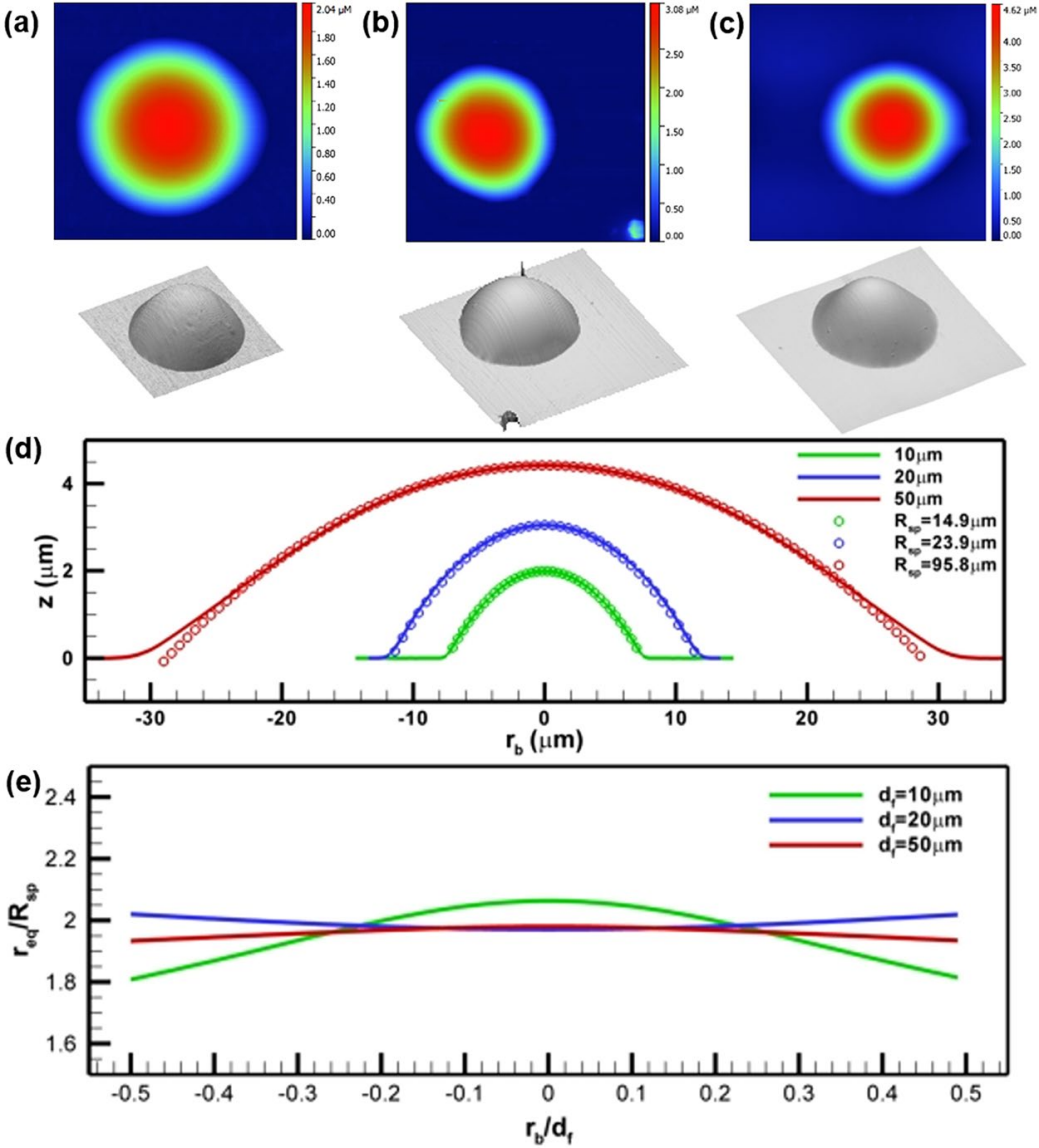
Asymptotic shape of a pico-liter oil drop with a circular base. 2D height distributions and its corresponding 3D rendering. Three sizes of base feature, *d*_*f*_, are measured at (**a**) 10, (**b**) 20, and (**c**) 50 *μm*. (**d**) Mean radial profiles (lines) of oil drop superimposed with its best fit profile of a sphere (symbols). Each profile is averaged over the azimuthal direction at the interval of 1°. The standard deviation of errors between best fit sphere (symbols) and measured profiles (lines) are determined at 0.0065, 0.2142, and 0.1056 *μm*. (**e**) Radial profiles of the local equivalent radius, *r*_*eq*_ = 1/*H*, normalized with radii of their corresponding spheres, *R*_*sp*_ = 14.9,23.9 and 95.8 *μm* respectively. The radial positions are normalized with the corresponding *d*_*f*_.

The sample profiles, *z*, of drops with circular contact lines are shown in Fig. 2a–c. With some of deviations, the contact lines are circular. The deviations are however expected since the micro-scale surface imperfections have much stronger impacts on contact line geometry. The 3D renderings show that the drop appears to assume the shape of a spherical cap. The mean radial profiles of drop (lines in Fig. 2d) are computed by averaging radial profiles over the azimuthal direction at the interval of 1°. These mean profiles are shown in the range of ±*d*_*f*_/2 to provide a clear cross-section view, where *d*_*f*_ is the characteristic length scale of the base. To test the hypothesis, we estimate the radius of sphere, *R*_*sp*_, using the best fit through data points located at the center portion of the profile as

1 $${R}_{sp}({r}_{b})=\frac{1}{2}\{\frac{{r}_{b}^{2}}{z(0)-z({r}_{b})}-[z(0)-z({r}_{b})]\},$$

where *r*_*b*_ is the radial position. The estimated radii of best fit spheres, *R*_*sp*_, are 14.9 ± 2.0, 23.9 ± 2.5, and 95.8 ± 4.0 *μm* for drops of 10, 20, and 50 *μm* respectively. Alternatively, the *R*_*sp*_ can also be estimated by a more generic form as

2 $${R}_{sp}=\frac{1}{2}[\frac{{A}_{c}}{\pi z(0)}+z(0)],$$

where *A*_*c*_ is the contact area. The estimated radii using Eqn. 2 are 15.1, 24.2 and 96.1 *μm*. In comparison, *R*_*sp*_ by Eqn. 2 is slightly larger than its counterpart by Eqn. 1, but identical in principle. For consistency, we will use the radii from Eqn. 1. The profiles of the best fit sphere (symbols in Fig. 2d) are superimposed on the measured profiles (lines in Fig. 2d) and match very well. The errors are determined as 0.0065, 0.2 and 0.1 *μm* for CIR10, CIR20, and CIR50 respectively. As one examines *r*_*eq*_ (Fig. 2e), the estimated *r*_*eq*_ is approximately 1.9 times of its corresponding *R*_*sp*_. Except for CIR10, the variations of *r*_*eq*_ over the entire drop are small. This clearly suggests that a pico-liter drop with pinned circular contact line must approach a spherical cap as it reaches equilibrium. Details are summarized in Table 2.

**Table 2:** Summary on geometric characteristics of a printed oil drop.

Feature	Height (*μm*)	Diameter (*μm*)	Volume (*pl*)	Sphere, *R*_*sp*_, (*μm*)	Bo (×10^−5^)	Oh *μ*/(*ρσd*_*f*_)^1/2^
CIR10	1.99	15.2	0.23	14.9 ± 2.0	1.23	0.8767
CIR20	3.05	24.4	0.703	23.9 ± 2.5	2.59	0.6199
CIR50	4.31	61.2	5.83	95.8 ± 4.0	10.6	0.3921
SQ10	3.06	14.3	0.24	9.85	1.27	0.8767
SQ20	4.01	29.0	1.137	28.3	3.57	0.6199
SQ50	4.44	58.5	6.0	98.5	10.9	0.3921

### Shape of drop with a non-circular contact line

**Figure 3:**
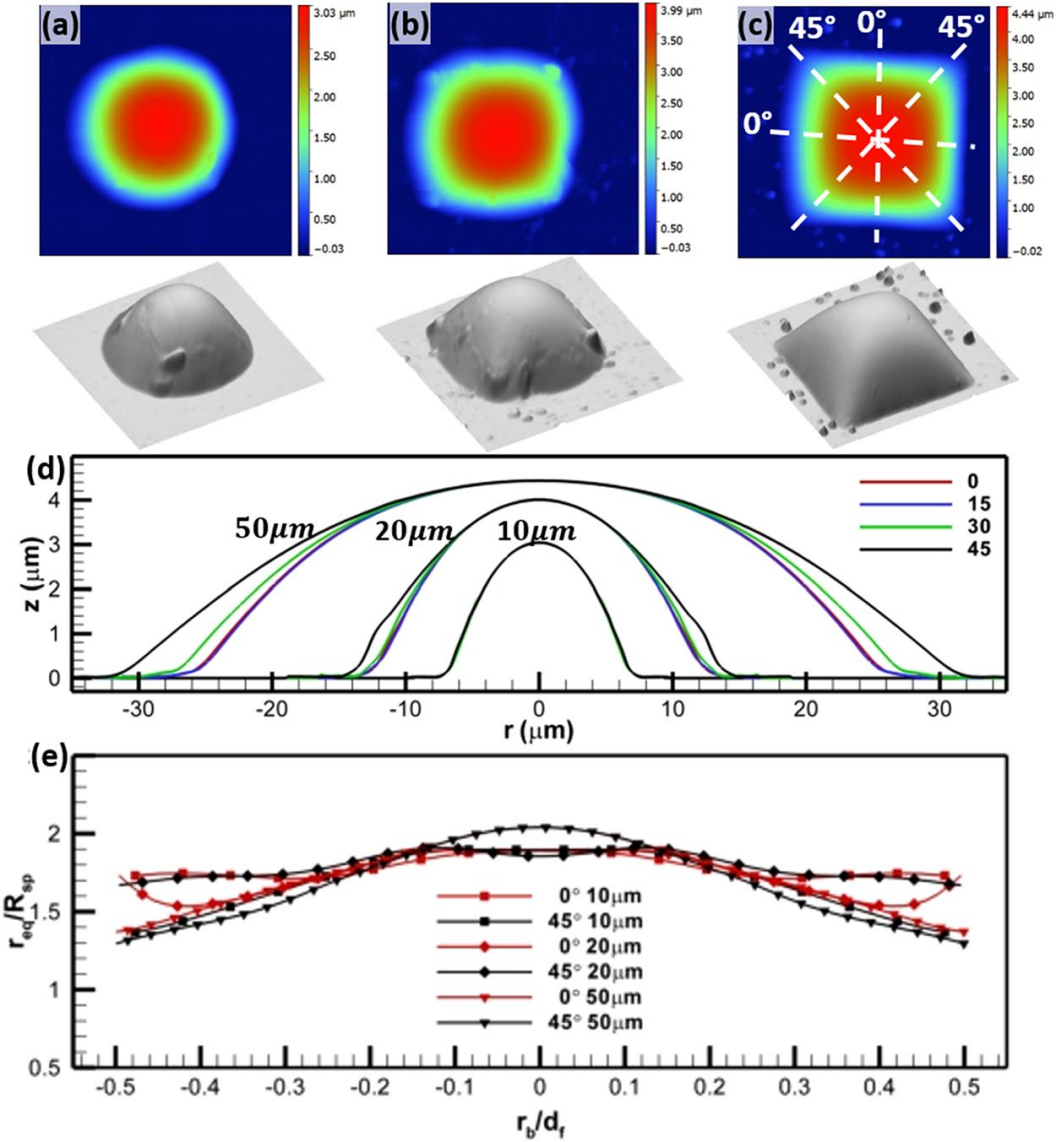
Asymptotic shape of a pico-liter oil drop with a square base. Row 1: 2D height distributions and its corresponding 3D rendering, Row 2: profiles at phase angles of 0, 15, 30 and 45, and Row 3: profiles normalized by base characteristics. The sampling phase angle is elucidated in **(c)**. The profiles at different sampling phase angles that are color coded. Three base feature sizes: (**a**) 10 × 10 *μm*, (**b**) 20 × 20 *μm*, (**c**) 50 × 50 *μm*, are used in the measurement and labeled in **(d)** and **(e**).

The sample profiles, *z*, of drops with a square base are shown in Fig. 3a–c. Deviations of the base from a square reduces as *d*_*f*_ increases, e.g. the 10 *μm* drop has an almost circular base, while the 50 *μm* drop has sharp corners. The 3D rendering in fact reveals that although at the smaller scale the contact line tends to have smooth corners, the imprints of sharp corners are still visible right above the base (Fig. 3a and b). The mean radial profiles of the drop (Fig. 3d) are averaged over radial profiles conditioned on their phase angles. The definition of phase angles is elucidated in the Inset of Fig. 3c, e.g. the 0° phase lines are those parallel to the sides and 45° phase lines are diagonals. The mean phase profiles of SQ10 are clearly collapsed suggesting the shape of a spherical cap similar to those of drops with circular base. As *d*_*f*_ increases, e.g. SQ20 and SQ50, the differences among mean phase profiles increase substantially as they approach the substrates, although there is a portion near the apex where phase profiles overlap. We estimate the radius of sphere, *R*_*sp*_, using Eqn. 2 as 9.85, 28.3 and 98.5 *μm* for SQ10, SQ20 and SQ50, respectively. The radial profiles of local *r*_*eq*_ normalized with their corresponding *R*_*sp*_ show a range that suggests constant *r*_*eq*_ located near the apex of the drop. It is rather evident that the black and red radial profiles with the symbol square and diamond are overlapping in the 60% center portion of the drops (Fig. 3e). However, the substantial deviation from a flat line are observed for SQ50. We speculate that SQ50 has not reached its equilibrium, of which we will establish in the following section.

Brief recap: we can conclude that the pico-liter drops have the asymptotic shape of constant mean radius of curvature averaged over two principle curvatures. It is evident that the difference in contact line energy can only affect the geometric shape, but the topography must have a constant mean radius of curvature. This is however mandated by Kelvin’s principle governing a curved liquid/vapor interface, since in the absence of inertia and other interfacial inhomogeneity the vapor pressure near the drop and Laplace pressure must remain constant.

### Time evolution of oil drop

**Figure 4:**
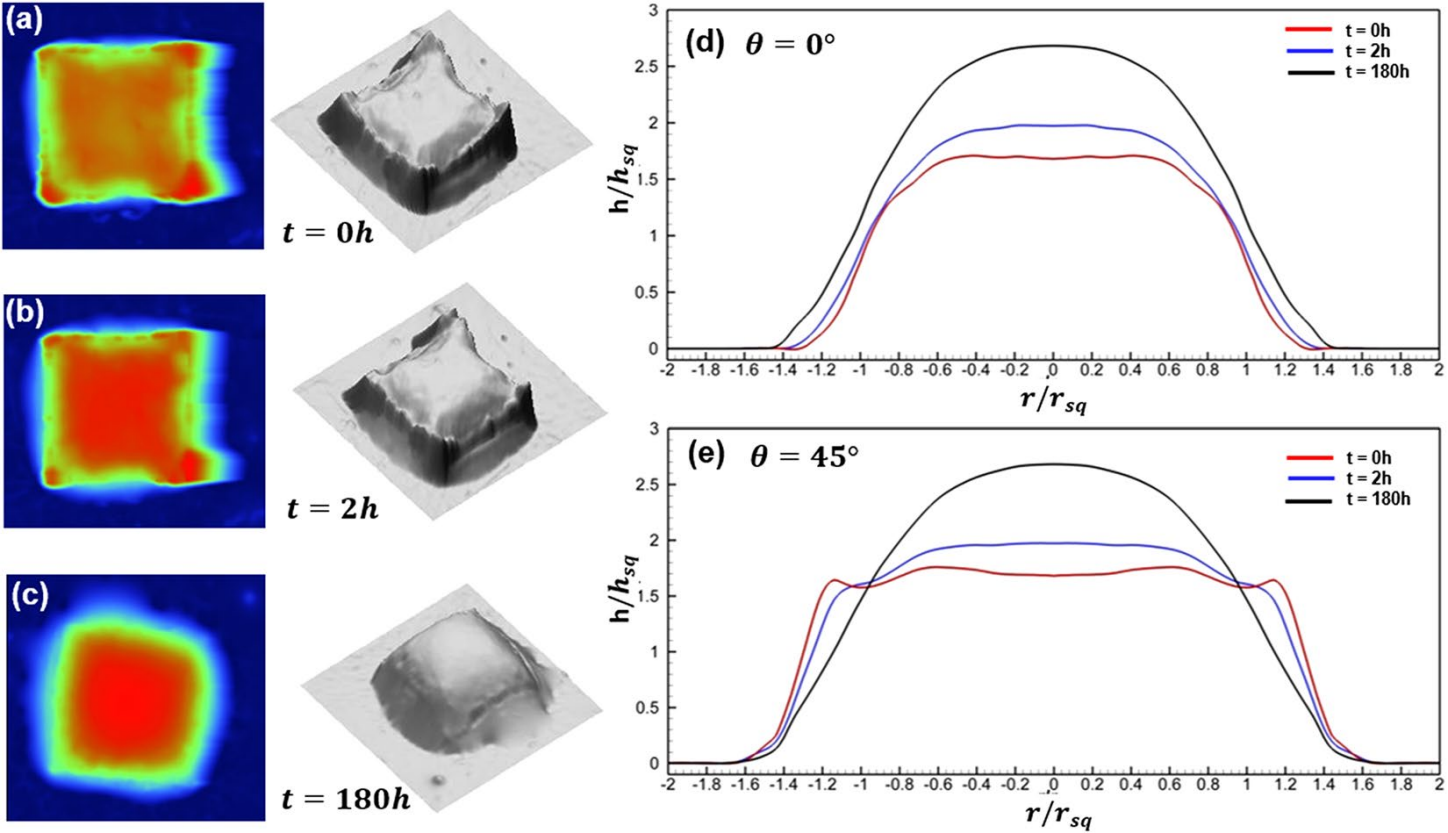
Temporal profiles of a pico-liter oil drop showing the relaxation of shape. Left: 2D height measurements by Atomic Force Microscopy; Middle: 3D rendering of micro oil droplets; Right: 2D cross-sectional profiles sampled at the phase angle of 0° and 45°. The evolution of droplet shapes is shown with AFM measurements obtained from the time immediately after printing to 180 hour afterwards, (**a**,**b**) *t* = 0 *h*; (**c**,**d**) *t* = 2 *h*; (**e**,**f**) *t* = 180 *h*. Cross-sectional profiles at phase angle (**g**) *θ* = 0; and (**h**) *θ* = 45 (diagonal), are shown to elucidate the evolution of the shape.

Although the “spherical” cap of a pico-liter drop is expected, a large number of drops with a non-spherical cap like top-hat have been observed. As time elapses, these non-spherical shaped drops will slowly relax to their asymptotic forms. However, the relaxation time scale increases substantially as the oil weathers. To further understand this phenomenon, we have visualized the evolution of an oil drop: the array of SQ20 oil drops is printed using the weathered crude (10 wt% loss after baking at 90 °C) where the surface tension remains unchanged but the viscosity increases significantly. Before peeling off the stamp, the prints with the stamp still attached are freezing at −20 °C for more than 8 hours. This additional procedure allows us to establish an initial drop shape in the geometry of the mold. Immediately after releasing the stamp, the time lapsed AFM measurements are performed at an interval of 15 minutes for the first two hours and then at an interval of 2 hours for eight days. The anecdotal result is summarized in Fig. 4. At t = 0 h immediately after peeling off the stamp, the drop (Fig. 4a,b) has a flat top with four sharp peaks at the corners. These peaks are the meniscus formed during the freezing. As the time progresses, the profile starts to relax and eventually reaches the shape of a smooth spherical cap (evident in cross-section profiles in Fig. 4d and e). Most noticeable is that it takes more than 7 days to reach its equilibrium shape. Note further that even at 180 hours the shape of drop has yet to reach its spherical cap. Further experiments using crude weathered at different levels ranging from 0% to 20% confirm our speculation that the relaxation time is prolonged with the increase of weathering.

Considering the possible mechanisms, we first note that the surface tension of the weathered oil remains largely unchanged between 15.5 mN/m for the fresh oil and 21.9 mN/m^45^ for 30% weathered oil, meanwhile the dynamic viscosity increases more than one order of magnitude from 10.1 to 141 *mPas*. Owing to its length scale, the inertia forces such as gravity do not have any significance in comparison to interfacial forces and properties, e.g. Bo ≪ 1, for all cases, where *Bo* is the Bond number (i.e. *Bo* = Δ*ρgh*^2^/*σ*_*LV*_, *h* is the characteristic height of the drop, Δ*ρ* the density difference between oil liquid and oil vapor, and *σ*_*LV*_ is the liquid-vapor surface tension), a dimensionless quantity comparing the hydrostatic pressure caused by gravity to surface tension. In the current scenario, without the hydrostatic pressure force as being present in millimeter drop, the de-wetting force is only balanced by the adhesion force; while the change of free-surface is determined only by interfacial forces including viscous forces. Hence, the temporal scale can only be affected by the interplay among interfacial force, inertia and viscosity, which may be elucidated by the dimensionless Ohnesorge number ($Oh=\mu /\sqrt{\rho \sigma {d}_{f}}$)^46^. Upon evaluating *Oh* for SQ20 printed with oil weathered to different stages, we have found *Oh* to be 0.62, 1.44, 2.5, and 7.28 for 0%, 10%, 20% and 30% weathered (by weight loss) oil respectively. We would like to emphasize that the abovementioned *Oh* are very large (in comparison, a 3 mm rain drop has *Oh* of 0.002), which suggests that the viscous force outweighs the inertia and surface tension at the interface of the pico-liter oil drop and leads to the long relaxation time.

### Non-Laplacian drop shape due to interfacial phenomena

**Figure 5:**
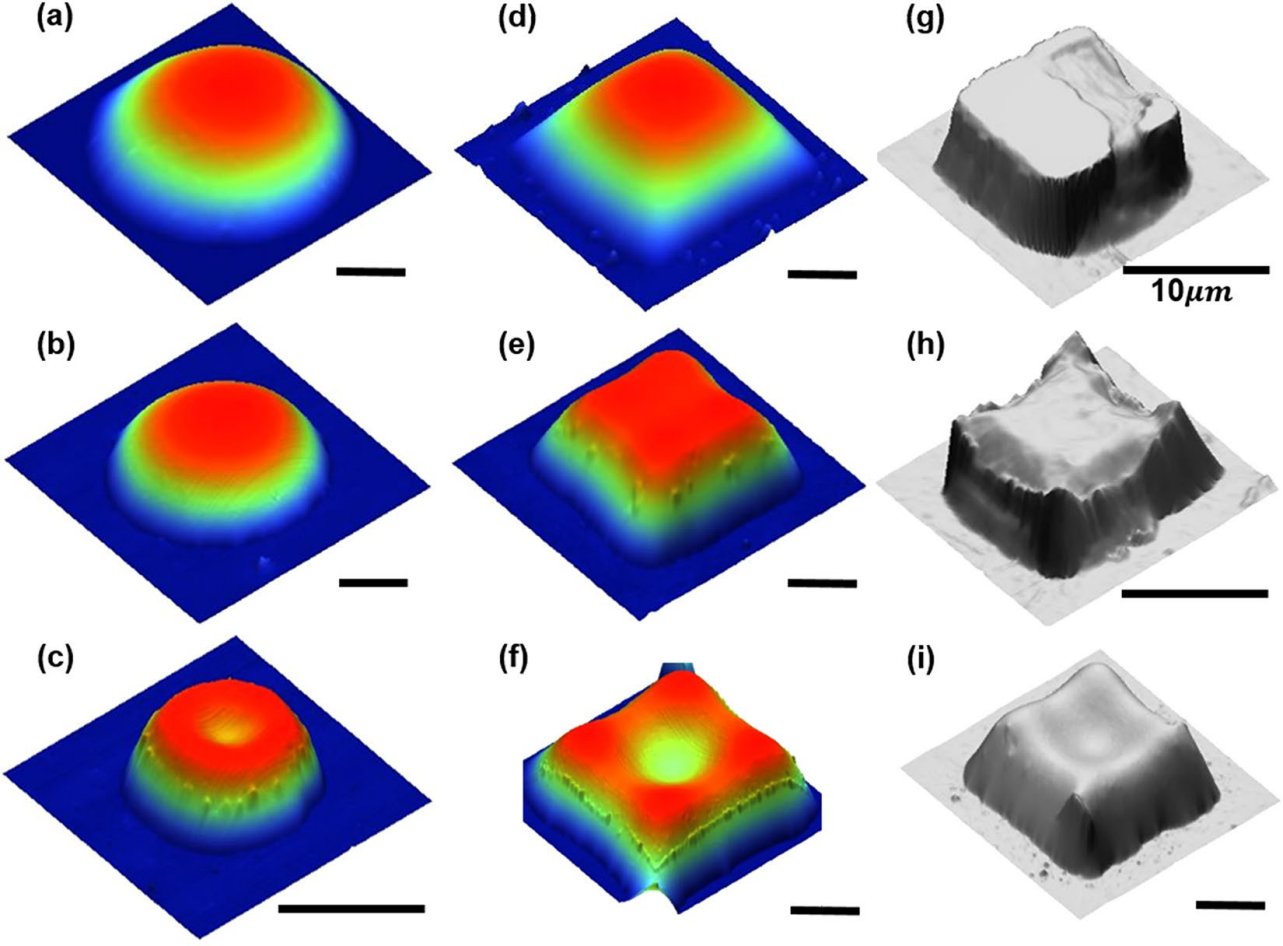
Assorted shapes of micro-drops. micro-drops with circular base of (**a**) dome, (**b**) top-hat, (**c**) donut profiles; micro-drops with square base of (**d**) dome, (**e**) top-hat, (**f**) donut profiles. The shapes are determined by interplay between interfacial forces and viscosity. The interfacial properties are adjusted by particle concentration and temperatures. (**g**–**i**) Micro-drops with irregular profiles initiated by freezing before releasing stamps. With low particle concentration, the viscosity and interfacial forces are affected primarily by temperature alone. Scale: 20 *μm* or otherwise specified.

As elucidated above, a pico-liter drop shape can only be affected by interfacial rheological properties, such as surface/interfacial tension, viscosity, and various interfacial processes, e.g. temperature and adsorbed particulates. To examine these effects, we have conducted an AFM survey of the shape of hundreds of drops over more than 100 prints generated under various conditions. We have observed drops with irregular shapes. Observations show that the majority of drops having caps with constant mean radius of curvatures (Fig. 5a and d) regardless *d*_*f*_ are printed with fresh or less weathered oil (<10%), and most importantly the interfaces of these drops are often pristine. In the absence of interfacial inhomogeneity, the pico-liter oil drop will reach equilibrium quickly. This relaxation process can be controlled by changing interfacial properties. We have observed a large number of drops with the flat top (Fig. 5b) when fresh crude at 0 C is used, under which condition, both surface tension and viscosity increase but Oh remains below unity. Note that the interface still remains clean. When weathered oil is used, i.e. similar surface tension, increasing viscosity and particulate concentration at the interface, most printed drops have flat top (Fig. 5e). It is also noted that the flat top is smooth and clean, but a small quantity of particles is accumulated at the edge of the flat top where the surface has the sharpest radial gradient (Fig. 5e). When the fresh oil containing a large quantity of particles is used, the printed drop results in an unexpected shape, e.g. donut shape (Fig. 5c and f). The nano-particles are also accumulated at the locations where contains the largest interfacial gradients. This may suggest additional processes, such as evaporation or dissolution, can also alter surface tension and nano-particle distributions at the interface. When these two effects combine, very irregular drop shape can be expected as evident in Fig. 5g–i.

### Robustness of texturing oily substrate with μTM technique

**Figure 6:**
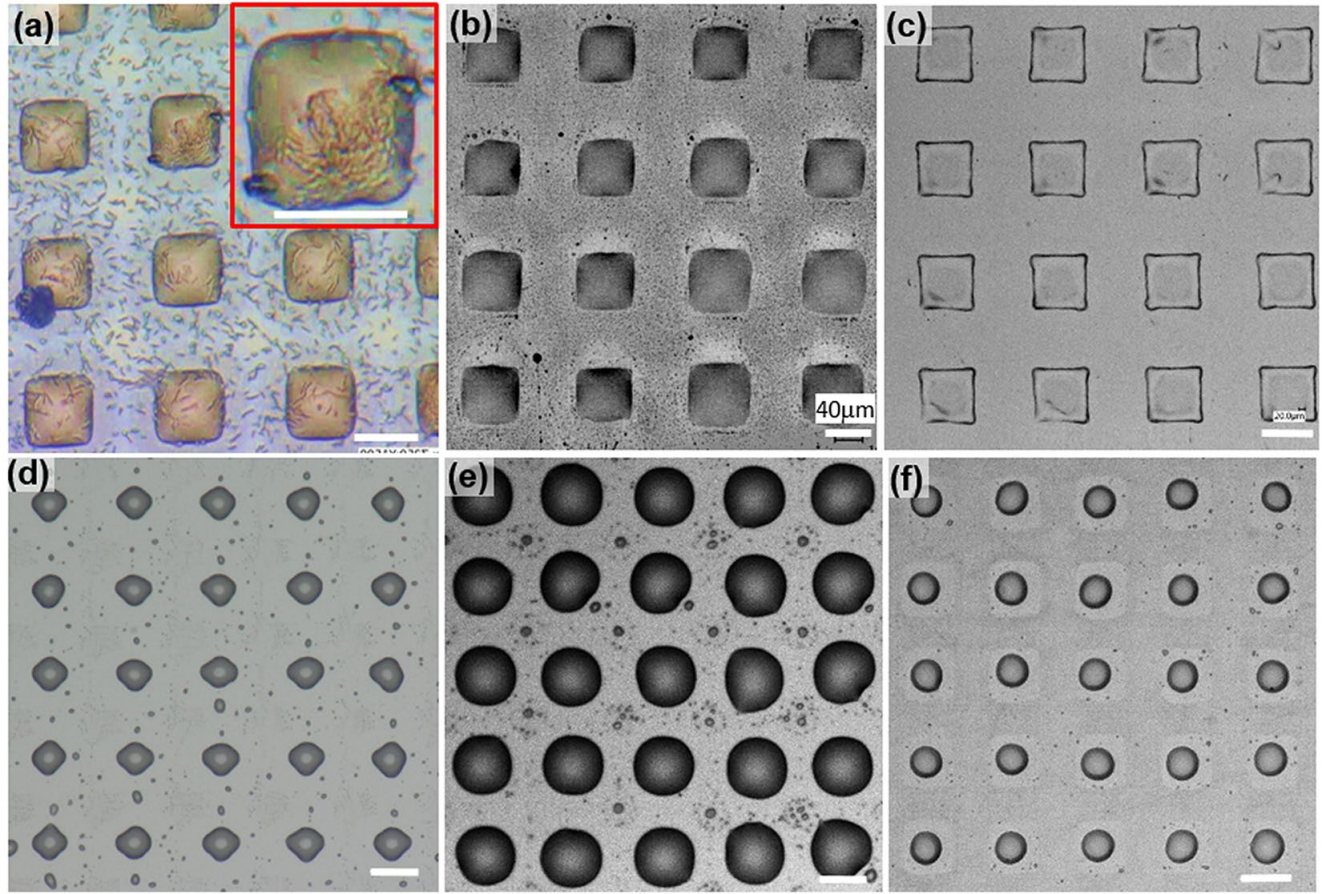
Pico-liter drops printed with: (**a**) crude oil, (**b**) 5W30, (**c**) 10W40, (**d**) 80% glycerol water mixture, (**e**) olive oil, (**f**) vacuum oil. Additionally, in (**a**), it is also shown the micrograph of crude oil drops after 96 hour growth of *Alcanivorex* culture at 30 C. Inset: Blowout micrograph showing bacterial film over a printed oil drops. Scale: 20 *μm* or otherwise specified.

To demonstrate the feasibility of the technique in biofilm studies, the printed substrates (Fig. 1) have been used to conduct preliminary growth experiment using *Alcarnivorex borkumensis*. During the experiment, the oily substrates were submerged in the medium and bacteria were allowed to grow at 30 C. The substrate was directly monitored by a Nikon TiE transmission microscope. The micrograph of the substrate after 96 hours growth is shown in Fig. 6a. Clearly, the drops retain their base shape and attach to the substrate, while the interactions between bacteria and oil-water interface are faithfully recorded. Note that the inset shows the preliminary “raft” building by bacteria. The topic of biofilm formation at oil-water interface is beyond the scope and will be communicated latter.

Exploring the range of applications, several oil substances have also been attempted. As shown in Fig. 6, besides crude motor oil, such as 5W30 (Fig. 6b) and 10W40 (Fig. 6c), have been successfully printed onto the substrate. The drops generated by these materials maintain their base shape and have similar 3D profiles. Other substances, not direct derivatives of petroleum hydrocarbon, such as 80% glycerol water mixture, olive oil and vacuum pump oil, are also successfully textured onto the glass substrate (Fig. 6d–f). However, these drops originally printed with square feature stamps cannot maintain the contact line with the sharp corners. The drops printed with 80% glycerol water mixture and vacuum oil have receding contact lines, whereas the drops printed with olive oil spreads over the substrate. Regardless of the irregularity in drop shape, the regularly spaced pico-liter drops are clearly printed.

It is also worth to point out that µTM methods in literatures are often limited by achieving neat feature detachment from stamps’ groves or wells and completely transferring to the printing substrate, which often leads to low yields in terms of the functional area. For solid materials such as metals, metal-oxides, and cured polymers, Carlson *et al*.^34^ has shown that the printing depends on the subtle interplay of interfacial energy systems, i.e. the energy of the printing material and stamp, as well as the one of the printing material and the printing substrate. To achieve successful transfer to the substrate from the stamp, the latter energy must exceed the former during the release of the stamp. Since the printing material in this work remains as a fluid during the printing, the printing processes are significantly more complex and discussion on its limitations remain qualitative based on anecdotal evidences. Although the base of a printed drop may not be limited in shape and size, the maximum volume of the drop is likely to be curtailed. Since the base of a drop is only determined by features on the stamp and governed by principles to cause spontaneous self-pinning of a contact line (discussed in great details in next subsection), one can in theory print an oil drop with a base of any shape and size. In practice, we have successfully printed oil drops with either square or circular base with sizes ranging from 5 µm to 100 µm. Anecdotal evidence is also provided in Fig. 1l that shows a clear print of letter “20” in the size of 0.5 mm. Note that the sharp corners of those letters are faithfully rendered. It needs to be pointed out and emphasized later (Materials and Methods) that the success in transferring a spatial pattern, i.e. an array of oil drops, is strongly affected by the inking of PDMS stamps. Apart from the need of completely filling wells/groves of the stamp, one must carefully remove the excess oil over the stamp surface, which after printing would otherwise form a liquid bridge to cause the merge of two nearby drops. Although any spatial patterns can be transferred in principle using the proposed µTM method, we have observed that there is some residue oil left in wells of the stamp after printing. It strongly suggests that there may be a maximum volume achievable for this method. Based on discussion about solid printing materials, one can speculate that the maximum volume for a printed oil drop with a fixed contact base depends on the interplays among interfacial energies at both oil-stamp and oil-substrate interfaces as well as capillary dynamics of oil trapped in each well during the release of the stamp. The maximum height of a drop we successfully printed in fact has not exceeded 5–6 *μm*. Since these interactions are complex and require substantial further experimentation and research, we will defer the detailed analysis to the future and later communications.

Although we have demonstrated the robustness of this method, the question on how does the printed drop maintain its non-circular contact line with sharp corners and non-Laplacian shape remains unclarified. In the following subsection, we will examine several possible mechanisms and propose a plausible explanation that could justify this unusual observation.

### Plausible mechanism for printing micro-drop with non-Laplacian shape

**Figure 7:**
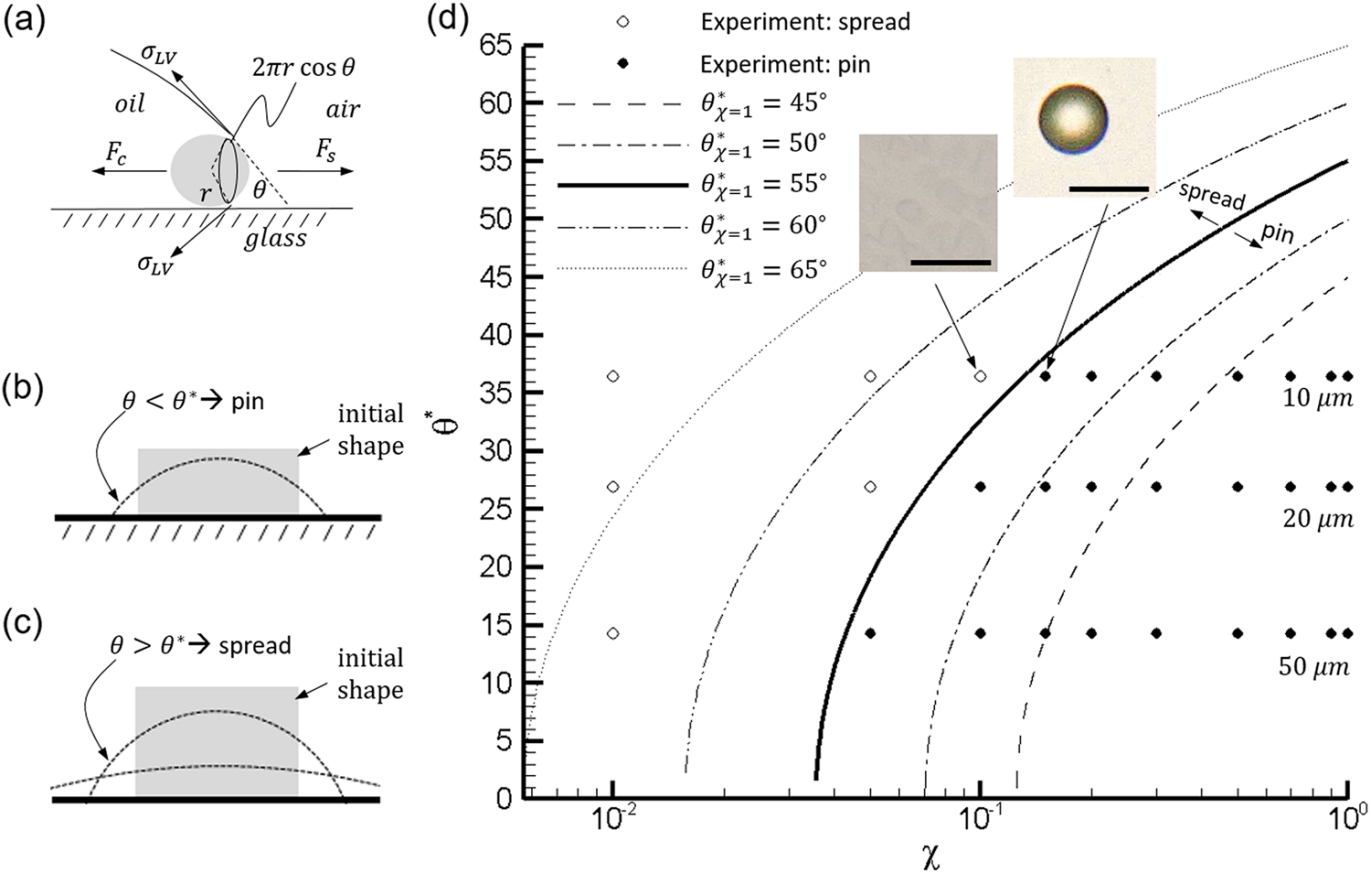
Mechanism of printing oily drops with spontaneous self-pinning of three-phase contact line: (**a**) schematic of the three-phase contact line with a confined particle is shown along with the spreading and capillary forces. In (**b**) and (**c**) are schematics for a pinned and spreading drop. In both (**b**) and (**c**) the gray rectangle is the initial drop after removing the stamp and the dashed lines are drop profiles shortly after. In (**b**) the apparent contact angle is less than θ* and the drop pins. In (**c**) the contact angle is greater than θ* and the drop spreads considerably. In (**d**) experiments that pinned (closed circles) or spread (open circles) using 10 *μ*m (*θ* = 36.4°), 20 *μ*m (*θ* = 27.0°) and 50 *μ*m (*θ* = 14.3°) diameter wells are plotted versus the volume percent *χ* of crude oil in hexadecane. The solid, dashed and dotted curves are plots of Eq. 6 assuming different values of the critical contact angle for 100% crude oil. The curves represent theoretical transitions from printable drops below the curve to unprintable drops above as annotated for the solid black curve. Two example images of a successfully printed drop and unsuccessful printing are inset in the figure where the scale bars are 10 *μ*m.

Brief recap: these printed micro-drops have (i) non-circular pinned contact line with sharp corners (Fig. 1); (ii) non-Laplacian drop shape with negative radius of curvatures (indents in drops as shown in Fig. 5c,f and i). Between these two, the pinning of the contact lines appears to be an initial but crucial step to determine the shape of the drop over a homogeneous substrate including non-Laplacian drop, whilst the latter may be explained as the following that (i) the surface energy presents complex landscape with many local minimums, i.e. different shapes, in the presence of a fixed boundary, and (ii) the transient states of a drop slowly evolving into its equilibrium state. In the first scenario, as the volume of a drop is large, the pinning stress at the contact line (inward towards the center of the drop) results in an increase in the internal pressure that leads to a convex surface; whereas a smaller drop with the same pinned contact line leads to outward stress at the periphery that results in lower pressure at the center of the drop and consequently a concave center. Although we do not have quantitative observations directly supporting this assertion, correlative evidence showing that those donut shaped drops often have smaller volume have indeed been observed. As for the second scenario, due to the lack of inertia, the drop shape evolves very slowly, often in days. Note that sample cross-section profiles of a drop evolving over seven days (Fig. 3) demonstrate the unexpectedly long relaxation time scale. In brief, it can be concluded that the pinning of the contact line supersedes any other processes during a print, hence, a key process that would allow those unconventional observations in this paper. In the following, we will use the concept of self-pinning of a contact line by nano-particles^47^ to provide a possible explanation for the abovementioned two observations. A series of experiments were designed and conducted to support our assertion on the contact line self-pinning by nano-particles (Fig. 7).

What are the possible means to pin the contact line of a pico-liter drop over a homogenous substrate? Conventional mechanisms on the pinning of a contact line often lie on (i) local imperfection or heterogeneity of the substrate; and (ii) altered interfacial properties due to the complex interactions between the crude to the substrate. However, it can be argued as the following that neither explanations appear to be mostly plausible. In the previous section, we have demonstrated extensively that printing crude oil drop was very robust. Hundreds of prints with different drop sizes and base shape have been consistently produced over many glass slides homogeneously functionalized with an OTS Self Assembled Mono-layer. Additional printing over non-functionalized glass slides was also proved possible but with a lower yield (e.g. ~50% of total printed area in comparison to 90% on an OTS treated substrate). This insensitivity to the substrate condition largely exclude the localized substrate imperfection from being the dominant cause of spontaneous contact line pinning. Although printing with crude was robust, we have found that printing with hexadecane (often used as a model oil system) was rather difficult. The printed hexadecane drops would quickly spread and merge with their neighboring drops to form a film of hexadecane upon the release of the stamp. Note that crude oil is primarily composed of alkanes such as hexadecane as well as aromatics and other hydrocarbons. Hence, the interfacial properties such as surface and interfacial tensions are not expected to differ substantially. Surface tension measurement (Fig. S1 and §S1 in SI) using a pendant drop setup^48^ shows that within the timescale of printing (ranging from 20 min to 2 hours), surface tension of crude reduces exponentially from 26.5 *mN*/*m* to 25 *mN*/*m* as particulates in crude congregate at the interface, while that of hexadecane remains at 24.5 *mN*/*m* (Fig. S1 in §S1). Although differing in values, it is not significant enough to affect contact line dynamics, which subsequently leads to our assertion that chemical interactions between crude and substrate is less likely the cause for spontaneous pinning of the contact line.

Examining the difference between crude and hexadecane in its physical composition, one can easily notice that crude oil contains a significant number of colloidal particles whereas hexadecane does not (as shown by Dynamic Light Scattering measurement in §S3 and Fig. S2 in SI that there is a clear presence of large number of nano-particles centered at 100 nm). This observation compounded by the robustness of the printing technique inspired us to reject the notion that the printing process may be only native to crude oil system but to seek an alternative but general mechanism that may work for other oily materials. Weon and Je^47^ have proposed a mechanism that would cause the self-pinning of a drop by colloidal particles confined at the contact line. As elucidated in Fig. 7a (also in^47^), when a particle is confined at the contact line it is expected to be partially wetted with an exposed cap of a perimeter 2*πr* cos*θ*, where *θ* is the apparent contact angle of the drop and *r* is the radius of the particle^47^. A capillary force exerted on each confined particle, 2*πrσ*_*LV*_ cos^2^*θ*, where *σ*_*LV*_ is the liquid-vapor surface tension, deters the spreading of the liquids nearby. As the number of particles confined at the contact line (*N*_*L*_) increases, the total capillary force, *F*_*c*_(=*N*_*L*_*πrσ*_*LV*_ cos^2^*θ*), increases linearly to counteract the spreading force, *F*_*s*_(=2*πRS*), where *S* is the spreading coefficient and *R* is the equivalent radius of the apparent contact line. The spreading coefficient, *S*, is often defined as *S* = *σ*_*SV*_ − *σ*_*LV*_ − *σ*_*SL*_, where subscript “*SL*”, “*LV*”, and “*SL*” denote the solid-vapor, liquid-vapor and solid-liquid interfaces, respectively^47,49^. Although the equilibrium spreading coefficient is invariable zero or negative, the “latent” spreading coefficient, *S*, for pure fluids can be positive^47^, which it is referred hereinafter as spreading coefficient for brevity. Additional force at the contact line such as drag force on the particles due to fluid motion may also be considered, but often deemed negligible owing to the slow flow between particle and solid substrate. To pin a contact line, the total capillary force, *F*_*c*_, must be balanced by or be greater than the spreading force, *F*_*s*_. This self-pinning model provides two consequential but relevant implications: (i) A drop can be possibly pinned over a chemically and topologically “homogeneous” substrate only by particles confined at the contact line; and its pinning effect varies with the concentration of particles, also defined in^47^ as a linear line packing fraction, *ϕ*_*L*_ = *rN*_*L*_/*πR*. At the critical condition when the contact line starts to pin (i.e. *F*_*c*_ = *F*_*s*_), a simple criterion for pinning can be established as

3 $${\varphi }_{L}^{\ast }=\frac{S}{\pi {\sigma }_{LV}{\cos }^{2}{\theta }^{\ast }}$$

where ${\varphi }_{L}^{\ast }$ is the critical (minimum) linear packing fraction for pinning and θ* is the corresponding critical (maximum) apparent contact angle. The model (Eqn. 3) demonstrates that by simply changing *ϕ*_*L*_ one can cause a normally de-pinning contact line to pin. (ii) The apparent contact angle of a drop can be altered by the concentration of those particles confined at the contact line and increases with proportion to *ϕ*_*L*_, as shown in the rewrites of Eqn. 3 as

4 $$\cos \,{\theta }^{\ast }={[\frac{S}{\pi {\sigma }_{LV}{\varphi }_{L}^{\ast }}]}^{\frac{1}{2}}$$

The abovementioned self-pinning model is particularly relevant in explaining the non-Laplacian shape of the printed oil drops, especially considering its pure physical and non-chemical specific nature. Elucidated and envisioned in Fig. 7b and c the shape of the printed oil drop may undergo the following changes: As the stamp is lifted off from the substrate, the drop would initially assume the shape of a printing well (e.g. shaded areas in Fig. 7b and c showing a profile of a top-hat cross-section). As the contact line spreads initially, it will be spontaneously pinned if the local apparent contact angle reaches below the critical contact angle, θ* (a scenario shown in Fig. 7b); otherwise it will continue to advance (shown in Fig. 7c). Note that the θ* increases with ${\varphi }_{L}^{\ast }$, i.e. the more particles adsorpted at the contact line, the larger the critical apparent contact angle will be. Considering a pinned pico-liter drop with a spherical shape when at equilibrium, the above observation implies that with the same printed base, more volume of oil can be printed if the contact line contains more particles.

To validate the self-pinning mechanism by particles at the contact line, we have designed and conducted the verification experiments. The objective of these experiments are to verify the following two conjectures: (i) The proposed mechanism would suggest that as one decreases the line packing density, *ϕ*_*L*_, (and consequently volume fraction, *ϕ*_*V*_) in oil, one would expect to observe the transition from pinned state to spreading of the printed droplet, i.e. for a given size of the printed oil drop, a critical *ϕ*_*L*_ (or *ϕ*_*V*_) must exist below which a printed drop cannot maintain its contact line and shape; (ii) Since the height of each printing well is maintained as a constant for different base features (e.g. 6 *μm*) and the equilibrium pico-liter drop assumes a spherical cap, the apparent contact angle for the printed and presumably pinned drop must increase with the decrease of its base contact area (i.e. a drop with the base contact area of 10 um in diameter has larger apparent contact angle than a drop with the base contact area of 50 um in diameter). Consequently the critical *ϕ*_*L*_ must also increase with the decrease of base contact area. In other words, a 10 μm print demands larger *ϕ*_*L*_ (or *ϕ*_*V*_) than a 50 μm print.

To test these two conjectures, we devised a binary system composed of a mixture of crude oil and hexadecane at various crude oil concentration, *χ*, defined as the volume fraction of crude in mixture. Justifications of using these two oily substances are that hexadecane contains no particles and a printed hexadecane drop will instantly spread, whilst crude oil contains large sub-micron and nano-particles and the printed oil drop will spontaneously pin on the substrate. Note that here we list the particle content of materials and their printing behavior at the same time, but without any inference to suggest their causal relationship. Additional justification can be attributed to their similar surface tensions (Fig. S1) and other comparable thermal dynamic properties. Owing to their chemical compatibility, the crude-hexadecane mixture allows us to systematically vary the particle volume fraction, *ϕ*_*V*_, with ease. One can relate the volume fraction, *ϕ*_*V*_, to linear packing fraction, *ϕ*_*L*_, as ${\varphi }_{v}=\pi {\varphi }_{L}^{3}/6$ based on the assumption of the homogeneous suspension. Further details are provided in Supplemental Material (S2). Therefore, the critical particle volume fraction for self-pinning can be described as

5 $${\varphi }_{V}^{\ast }=\frac{\pi }{6}{(\frac{S}{\pi {\sigma }_{LV}})}^{3}{\cos }^{6}{\theta }^{\ast }$$

The surface tensions of crude oil and hexadecane are essentially equivalent (Table S1). We have performed additional surface tensions measurements using a pendent drop experimental setup and analysis^48^. It is found that crude and mixtures of crude and hexadecane see their surface tension decrease with time, but this change occurs on the order of tens of hours (see supplementary material). Therefore, at the relevant printing time scale (<10 min), it is reasonable to assume that *σ*_*LV*_ is constant regardless of *χ*. Additionally, the chemical makeup of the bulk of crude oil and hexadecane are fairly similar and the substrate material does not change. This along with the evaporation rates of both crude and hexadecane being negligible leads us to speculate the spreading coefficient *S* is also a weak dependency of *χ*. Thus, the particle volume fraction depends only strongly on apparent contact angle, ${\varphi }_{V}^{\ast }=f({\theta }^{\ast })$. Since direct measurement of *ϕ*_*V*_ of crude-hexadecane mixture is not readily available, but with a reasonable assumption that *ϕ*_*V*_ of the mixture is linearly proportional to crude oil volume fraction, *χ*, as *χ* = *ϕ*_*V*_/*ϕ*_*V*,*χ* = 1_, where *ϕ*_*V*,*χ* = 1_ is the particle volume fraction for crude, we can thus establish a relationship as

6 $${\chi }^{\ast }={(\frac{\cos {\theta }_{1}^{\ast }}{\cos {\theta }_{\chi }^{\ast }})}^{6}$$

where ${\theta }_{1}^{\ast }$ is the corresponding critical contact angle for ${\varphi }_{V,1}^{\ast }$.

To test these conjectures, we print the array of drops with crude-hexadecane mixtures. We systematically vary the crude concentration, *χ* and subsequently particle volume fraction, *ϕ*_*v*,*χ*_, to determine a critical particle concentration, below which printing is not possible. The volume concentrations, *χ* = 0.9, 0.7, 0.5, 0.3, 0.1, 0.05 and 0.01, are used for printing 50 μm circular based (CIR50) drops. It is found that when *χ* ≥ 0.05 printing is possible, while at *χ* = 0.01 the drops spread (Fig. 7d), hence the critical volume concentration, χ^*^ ≈ 0.05. When we reduce the base feature size of drops from 50 μm down to 20 μm (CIR20) and 10 μm (CIR10) drops, this trend persists and agrees very well with the conjectures, i.e. above certain concentration, *χ*, the printed drops pin; while below it they spread. The results are summarized in Fig. 7d and Table 3. Note that the critical volume concentration, *χ*, increases as the base feature size decreases, i.e. for CIR10, 0.10 < *χ* < 0.15, and for CIR20, 0.05 < *χ* < 0.10, while for CIR50, *χ* reduces down to between 0.01 and 0.05. This observation in fact supports the second conjecture: as the particle concentration (*ϕ*_*v*_) increases, the contact line can support a drop with larger apparent contact angle. Note that the apparent angle for a pico-liter sessile drop that assumes the shape of a spherical cap depends on the total printed volume. For a stamp with a constant well depth, they will result in larger apparent contact angle for a smaller printing base (e.g. CIR10) than that for a larger base (e.g. CIR50). To maintain its base, higher particle concentration is needed for a drop with a smaller base. Since we do not have the direction measurement of the particle concentration of crude, ${\varphi }_{V}^{\ast }$, and consequently the maximum critical apparent angle, ${\theta }_{1}^{\ast }$, we have plotted relation (Eqn. 6) with several prescribed parameters varying from 45° to 65° at an interval of 5°. It can be seen in Fig. 7 that the regime boundary suggests ${\theta }_{1}^{\ast }$ of 55°, i.e. for crude oil drop, the self-pinning mechanism by nano-particles can hold the drop with the apparent contact angle up to 55°. These observations clearly match with our conjectures above and support our assertion that the ability in micro transfer printing of oil with non-circular base is owing to the self-pinning of contact line by nanoparticle adsorption along it. This result suggests the robustness of the technique using micro-transfer printing to structure a substrate with colloidal suspension.

**Table 3:** Nanoparticle line packing density and base size of the drop affect the printability.

*χ*	CIR10	CIR20	CIR50
0.01			spread
0.05		spread	Print
0.10	spread	print	Print
0.15	print	print	Print
0.20	print	print	Print
…	…	…	…
1	print	print	Print

Contact line packing density can be determined by volume fraction of crude in the crude-hexadecane mixture, *χ*. Details are presented in SI §S2.2–S2.3. The experiments are performed using three different base size: 10, 20 and 50 *μ*m. The base feature is circle. The printability is characterized as: upon the release of the stamp, “Print” indicates a printed drop can maintain its base shape and size, and “Spread” suggests otherwise.

### Wetting property of the printed oily substrate

To achieve the original objective to study bacterial interactions and biofilm formation at oil water interface, the wetting property of the textured substrate needs to be quantified. We have measured static contact angle and its hysteresis using an in-house developed tilting plate goniometer. Although three water drop sizes, 10, 15 and 20 μl, were used in this measurement, we here only present 20 μl results for brevity. The results are summarized in Table 4. It can be shown that the oily textured surfaces are uniformly hydrophobic but have large hysteresis.

**Table 4:** Static water contact angle (CA) and Contact Angle Hysteresis (CAH) for various textured substrate.

Feature	Pattern	CA	CAH
SQ5	Regular	114	57.5
SQ10		107	53.7
SQ20		103	52.3
SQ50		103	53.7
SQ10	Checker Board	106.3	58.3
SQ20		109.7	62.7
SQ50		109.7	62
CIR10	Regular	104.7	54
CIR20		102.3	58.4
CIR50		105.7	56.7

## Conclusion

In this paper, we have demonstrated that the technique, micro transfer molding (μTM), is an effective means to texture a smooth substrate with a massive array of pico-liter oil drops. These textured surfaces provide crucial platform for mechanistic studying on bacterial interactions with oil, which allows us to observe individual bacterial motion at the oil water interface as well as to quantify the population response of bacterial suspensions such as particle dispersion, chemotaxis, and adsorption to the oil water interface.

In the paper, we have extended µTM technique to transfer oil substances include crude oil onto a chemically and topologically homogeneous smooth substrate and textured it with precisely controlled micro-drops. We have shown that with crude oil the pico-liter drops gave been successfully printed. The drops have different sizes ranging from 5 to 50 *μm* and different base shapes including those with sharp corners. Owing to their small size, these drops are very robust in maintaining its base shape even those with sharp corners and adhering to the substrates under various environmental conditions, which allow us to achieve our objective of understanding biofilm formation at oil water interface. Using AFM, we have characterized the shape of drops with different sizes and base shapes. It is found that regardless of variations in base shapes and sizes, the asymptotic shape of the printed drops is invariantly a “spherical” shape of constant mean radius of curvature. With the maximum height of a few micrometers for each drop, the inertia forces are negligible in comparison to those interfacial forces (i.e. Bo ≪ 1). It can be argued that the Laplace pressure of a printed pico-liter drop is constituted primarily by the actual vapor pressure near and saturation pressure within the drop. The hydrostatic pressure, that is dominant in a micro-liter drop, is not critical in determining the shape of a pico-liter drop. Consequently, the pico-liter drop with pinned contact line must have the asymptotic shape of constant mean radius of curvatures.

Additionally, owing to its small size, a pico-liter drop with pinned contact line takes much longer to reach its equilibrium shape than their micro-liter counterparts. Using 10% weathered oil as the printing medium, we have demonstrated that at micro-scales drops reach their “spherical” equilibrium shapes from the initial “top-hat” shape in almost seven days. This phenomenon can be attributed to the subtle balance between the interfacial force and oil viscosity. This point can be clearly demonstrated in the comparative experiments, in which the drops with weathered oil having the same surface tension but much larger viscosity than the fresh oil reached the spherical shape in days compared to merely a few minutes. Further characterization experiments reveal that drops with convex topology can also be generated with careful manipulation between surface tension and viscosity by adsorbing particles or by material properties.

The developed technique has been applied to texture a smooth substrate for studying the biofilm formation at the oil water interface. The preliminary results on bacteria rafts built by *Alcarnivorex borkumensis* over the pico-liter oil drops demonstrated the robustness in structuring surface and the suitability in biophysical microbial studies. The details on bacterial interactions near these interfaces will be reported in the later communication. Additionally, the technique has been applied to different oil and successfully structured the substrate with them, which demonstrates the robustness of the proposed technique in performing micro-scale material transport over these textured colloidal surfaces.

## Materials and Methods

### Fabrication of stamps for micro transfer molding

**Figure 8:**
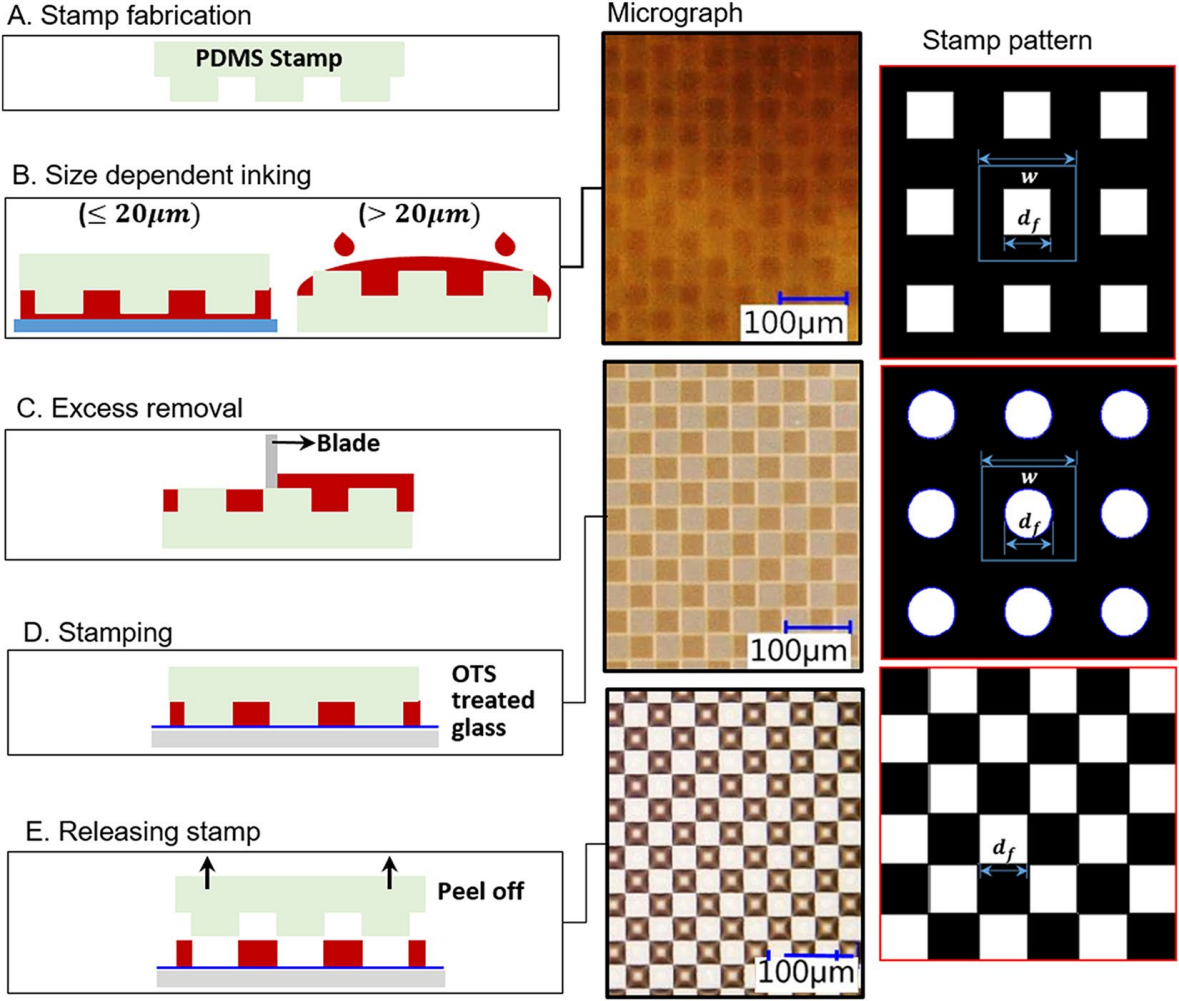
Surface functionalization procedures using micro transfer molding. Left: Graphical illustration of fabrication procedure. Middle: micrographs showing the corresponding state of the stamp and surface. Right: Characteristic pattern of stamp.

The polydimethylsiloxane (PDMS) stamp with desired feature and depth is replicated from a positive silicon master with appropriate micro-structures. Step A in Fig. 8 shows the side-view schematics of a PDMS stamp. The characteristics of the patterns are summarized in the right panel of Fig. 8 and their dimensions in Table 1. Note that the wells on the (negative) PDMS stamps correspond to the posts or pillars on the (positive) silicon master.

The silicon master is produced by conventional microfabrication techniques: the micro-pattern is first transferred to a 4″ n-type 〈100〉 silicon wafer from a chrome mask (right panel in Fig. 8) using photo-lithography^50^. After patterning, the wafer is then etched with Bosch Deep Reactive Ionic Etch (DRIE) process (Plasma-Term SLR-770) to create positive micro-structures (e.g. circular and square micro-posts) with a depth of 6 *μm* over the wafer. Alternatively and most commonly, the master can also be created out of photoresists directly, e.g. SU8-2050. The disadvantage is the possible delamination of features after several replications. Each stamp has an effective area of 3 × 3 *cm*^2^ that contains micro pillars with appropriate features (Table 1). The features are arranged in the spatial configuration of regular, staggered and checkerboard patterns. The polymer stamps are replicate-molded from Si masters. Sylgard 184 silicone elastomer (Dow Corning, Inc) is thoroughly mixed with the curing agent at a ratio of 10:1. After degassing, the mixture is casted onto the master and cured at 80°C for 120 mins. The thickness of the stamps is maintained less than 2 mm to ensure the flexibility of the stamps that is important to properly transfer the oily micro-droplets onto a flat substrate. The replicated stamps are peeled from Si masters and washed consecutively in acetone, methanol, isopropyl alcohol and deionized water solutions. The stamps can be re-used after the same wash protocol is applied.

### Surface treatment of substrate before printing

The substrates are microscope slides. It is first cleaned by “piranha” etching solution (98% H_2_SO_4_ and 30% H_2_O_2_ at the ratio of 2:1 v/v) at 25 °C for 90 min. After rinsing thoroughly in deionized water and drying with nitrogen, the substrate is baked on a hot plate at 150°C for 50 minutes. The substrate is then treated with oxygen plasma for 30 seconds (200 sccm O_2_ at 200 W), and functionalized homogeneously with OTS (octadecyltrichlorosilane) monolayer by submerging the substrate in the solution (0.2 v/v % OTS in toluene) for 20 min at 25 °C. The OTS-coated slides are rinsed with toluene, dried with N_2_ and baked at 90 °C for 30 min to form a covalent bond between the substrate and the polar head (SiCl3-) of the amphiphilic OTS molecule [CH_3_(CH_2_)_17_SiCl_3_]. The glass is then washed with the abovementioned protocol and dried with nitrogen. Note that the printing surface is homogeneously functionalized without pre-arranged patterns. With alkyl group facing outward, the glass slide is intrinsically hydrophobic (with a water contact angle of 110°). Several other substrates, such as untreated and O_2_ plasma activated oxidic glass, are also tested for the yield of printing micro oil drops in conjunction with the OTS treated surface. It is believed that with the alkyl group the crude may adhere to the OTS-substrate stronger than to untreated and oxidic substrates, which leads to better preservation of printed features. It needs pointing out that the differences in printing quality are in fact subtle. But the concept of promoting adhesion of oil drops on OTS substrate is attractive and inspires the following printing steps.

### Printing pico-liter oil drops using Micro Transfer Molding (*μ* TM)

We have employed a variant of Micro Transfer Molding (*μ*TM) technique^51^ in combination of Micro Transfer Printing techniques^34^. Instead of transferring curable, the method is to transfer crude or oily fluids to form array of “sessile” micro drops over a homogeneous substrate, in order to create the substrate for studying complex interaction and biodegradation processes by bacteria near oil water interface. Louisiana light crude is used here. Prior to inking, crude is heated in a bath at 80 °C to reduce its viscosity and surface tension. For smaller features (≤20 µm), the oil is inked using capillary interactions over a thin oil film spin coated on a wafer; for larger feature sizes (>20 µm), oil is infiltrated via gravidity by placing oil directly on the stamp (Step B in Fig. 8). The excess oil must be removed by the edge of a razor blade (Step C in Fig. 8). Note that Step C is vital to maintain the integrity of a single feature and to prevent nearby drops from connecting after printing. The stamp is then brought into contact with the OTS coated substrate (Fig. 8D) and slowly peeled off after 10 min (Step E in Fig. 8) to reveal the pattern (micrograph in Fig. 8A). The middle panel graphically shows states of the stamp and substrate during printing a checkerboard pattern of 50 *μm* square. It is clear that stable colloidal oily pattern can be established. Assorted results (Fig. 1) will be discussed later in details. Additional oily materials have also been successfully printed. The key properties of oils used are summarized in Table S1.

### Atomic Force Microscopy (AFM) measurement

To quantify the shape of pico-liter drop and to elucidate mechanisms in forming these drops, Atomic Force Microscopy (AFM, Dimension 3000) is used. With the superior lateral resolution, AFM has increasingly become the go-to technique for measuring topology and assessing interfacial forces of micro-drops^52–55^. Since crude has a relative high surface energy density (*σ*_*lv*_ > 26 *mJ*/*m*^2^), it allows stable imaging by AFM. Additionally, the relatively low vapor pressure at room temperature (~28 kPa) for Louisiana light crude ensures no noticeable evaporation during the time scale of experiments (~20 min). Furthermore, since less than 6 wt% of the crude used is poly aromatic hydrocarbon (PAH), the evaporation will not be significant. This point can be elucidated from Scanning Electron Microscope (SEM) measurement (Fig. 1h) of an array of 20 um square oil drops. Since we focus primarily on the shape of the drop, constraints for AFM measurement are significantly lessened.

In current experiments, AFM was operated at tapping mode using a monolithic Si tip with a tip radius ≤10 nm. The oil drops to be imaged were selected randomly to provide sufficient coverage over the entire print. The topography of three different drops per print were measured. The scan rate of the AFM scanner was maintained between 0.35 and 0.9 Hz with maximum 128 samples/line. It was determined experimentally that this low scan frequency was critical to prevent the probe from disturbing the drop interface and create structures such as striations and wavy surface topology (ref.^55^). A sample AFM measurement showing the array of 50 *μ*m square oil drops was provided in Fig. 1d. It was also observed that with the fast scanning speed, the drop profiles were elongated in the scanning directions. Such imaging defects can be mitigated using slower scanning speed and lower imaging force.

### Measurement of local mean curvature

Local principle curvatures of drop are calculated by locally curve fitting a second-order polynomial surface over AFM measured drop surface (analysis procedure shown in SI §S4). The local mean radius of curvature is then directly computed to characterize the shape of each printed drop.

### Macro surface characterization using water contact angle

The wetting property of the oily textured surface was characterized with static and dynamic water contact angle as well as the durability under shear flow conditions. The water contact angle measurements were performed by an in-house developed goniometer that includes a high speed CMOS 1 K × 1 K camera (IDT-N3) operating at the rate of 1,000 frames/sec (fps), a 2-axis translational stage, and a monolithic light source. All components were mounted on a breadboard attached to a 3-axis rotational platform allowing dynamic contact angle measurements. Contact angles were measured at ambient temperature. Water drops of 10, 15 and 20 µl were used for the measurement. During the dynamic contact angle measurement, the platform was rotating gradually from horizontal (0°) to vertical (90°) positions while the high speed images of the drop profile were recorded and stored for image analysis.

## Electronic supplementary material

Supplemental Information
