# Supplementary material for: Fabrication and characterization of a scalable surface textured with pico-liter oil drops for mechanistic studies of bacteria-oil interactions
Source: Sci Rep. 2018 May 15;8:7612. doi: 10.1038/s41598-018-25812-y (PMC5954110; doi:10.1038/s41598-018-25812-y)
Supplement: Supplementary file 1 — Supplemental Information [file 41598_2018_25812_MOESM1_ESM.pdf]

Supplemental Information: Fabrication and characterization of a scalable surface textured with pico-liter oil drops *by Jalali, White, Marti and Sheng*

S1. Measurement of Surface Tensions

The pendant drop method [1] is used to measure the surface tension between air and crude oil as well as air and 5% (v/v) crude in hexadecane. The surface tension measurements over time are shown in Fig. S1. The 5% crude-hexadecane mixture is measured for 8 hours when the surface tension has reached its equilibrium at about 24.5 mN/m, whilst the surface tension of crude is only measured for three hours due to the drop detaching from the needle at the end of experiment. Upon detachment, the surface tension of crude-air is approximately 21.75 mN/m that is much lower than that of 5% crude-hexadecane mixture. However, the two systems have similar initial surface tensions; the surface tension of 5% crude hexadecane mixture starts at 26.5 mN/m in comparison to that of crude at 27 mN/m. As time evolves, the surface tension of both liquids reduces exponentially. This trend can be attributed to the adsorption of oleophilic particles within crude onto interface that reduces surface energy. The decay rate decreases with the particle

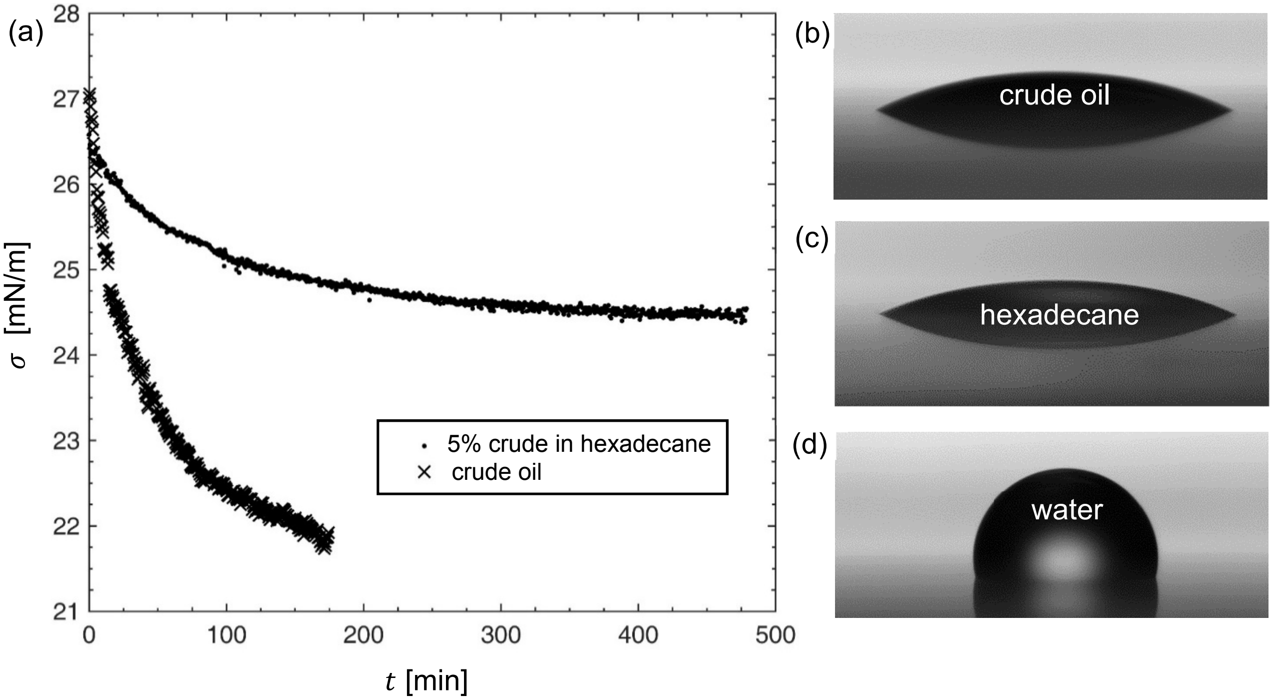

Fig. S1| In (a) the surface tension of a crude – air and 5% crude in hexadecane – air surface using the pendant drop method is plotted versus time. In (b) and (c) 5 μl sessile drops of crude oil and hexadecane, respectively, are shown on OTS-coated printing substrate. The surface is clearly oleophilic with comparable apparent contact angles for crude and hexadecane drops. For comparison (d) showcases a water drop over the same substrate showing hydrophobic characteristics.

concentration, i.e. 5% crude-hexadecane mixture has higher equilibrium surface energy than 100% crude. Although the decay rates and equilibrium surface tensions differ substantially with respect to the volume concentration of crude, the surface tensions within the printing time scale (e.g. < 10 min) remain largely the same. (i.e. 25.5 mN/m for crude and 26.25 mN/m for mixture). Therefore the difference in surface tensions for the crude and crude-hexadecane mixtures within the time scale of printing vary only about 1%, and the changes in surface tension are less likely to cause spontaneous contact-line pinning. Additionally, macro sessile drops over the printing substrate (e.g. OTS treated glass) of crude and hexadecane show similar contact angles. This observation provides comforts in our assertion that contact line pinning is less likely resulted from intrinsic material properties.

S2. Model for Self-pinning Drops by Particles at Contact Line

S2.1 Brief description on model.

When a particle is adsorbed at the contact line (in Fig. 9a and [2]), a capillary force will be exerted on the liquid film to counteract the spreading of the drop (or the contact line). When the number of particles is sufficiently large, the capillary force is large enough to pin the contact line completely. This self-pinning by particles can be best elucidated graphically as Fig. 9a and described mathematically as

$$\cos^2\theta = \frac{S}{\pi\sigma_{LV}\phi_L}, \tag{S1}$$

where $\phi_L = rN_L/\pi R$ is termed as the line packing fraction, a dimensionless number indicating the fraction of the contact line with equivalent radius of R occupied by solid particles of mean radius of r. Here N_L is the total number of particles at the contact line, S is the initial spreading coefficient, and σ_{LV} is the equilibrium surface tension between liquid and vapor. With a fixed line packing fraction, ϕ_L^*, relation S1 predicts a maximum apparent contact angle, θ^*. If the apparent contact angle of a drop exceeds θ^*, the contact line will move; if the angle is less than θ^*, the drop will be pinned.

S2.2 Relate line packing fraction, ϕ_L, at contact line to volume packing fraction, ϕ_V

The relation (Eqn. S1) can be rewritten in terms of volume packing fraction, ϕ_V, a dimensionless quantity representing the fraction of the total volume occupied by particles. One can define the volume packing fraction as

$$\phi_V = \frac{\frac{4}{3}\pi r^3 N_V}{V}, \tag{S2}$$

where V is the volume of the drop, and N_V is the number of particles contained within the drop. Assuming a spherical cap of a pico-liter drop with contact line radius of R, the apparent contact angle, θ, is related implicitly to droplet volume, V, as the following

$$V = \frac{\pi R^3 (2 - 3\cos\theta + \cos^3\theta)}{\sin^3\theta} \tag{S3}$$

To relate ϕ_L to ϕ_V we must relate N_L to N_V. We utilize the relation that the number concentration (i.e. number of particles per unit volume), $n_V = \frac{N_V}{V}$, is simply as

$$n_V = n_L^3,$$

where n_L is the number concentration per unit length and can be approximated as $n_L = \frac{N_L}{2\pi R}$. Hence, we obtain N_V in terms of N_L as $\left(\frac{N_V}{V}\right) = \left(\frac{N_L}{2\pi R}\right)^3$. Recalling that $\phi_L = rN_L/\pi R$, the above relation can be expressed in terms of ϕ_L,

$$\left(\frac{N_V}{V}\right) = \frac{1}{8}\left(\frac{\phi_L}{r}\right)^3 \quad \text{or} \quad \frac{r^3 N_V}{V} = \frac{1}{8}\phi_L^3.$$

Substituting the above relation to Eqn. S1, we relate the ϕ_V with ϕ_L as

$$\phi_V = \frac{\pi}{6}\phi_L^3. \tag{S4}$$

Hence, the relation between apparent contact angle and line packing fraction can be obtained by substituting Eqn. S4 into Eqn. S1 as the following form

$$\cos^2\theta = \left(\frac{S}{\sigma_{LV}}\right)(6\pi^2\phi_V)^{-\frac{1}{3}} \tag{S5}$$

or the more intuitive form:

$$\cos^6\theta = \frac{1}{6\pi^2\phi_V}\left(\frac{S}{\sigma_{LV}}\right)^3. \tag{S6}$$

The relation (Eqn 6) show a power law dependency between the apparent contact angle, θ, and volume packing fraction, ϕ_V.

S2.3 Model for crude-hexadecane binary system

Since direct measurement of ϕ_V is difficult, we resort to using a crude-hexadecane binary system to vary the volume packing fraction, ϕ_V, by changing the volume concentration of crude in crude-hexadecane mixture. The resultant, $\phi_{V,\chi}$, is

$$\phi_{V,\chi} = \frac{\phi_{V,1} \cdot V_{crude}}{V_{hexadecane} + V_{crude}} = \chi \cdot \phi_{V,1}, \tag{S7}$$

where χ is the volume concentration of crude in mixture, $\chi = \frac{V_{crude}}{V_{hexadecane} + V_{crude}}$, assuming simple volume addition to the mixture. Therefore, the model (Eqn. S6) can be expressed in terms of χ as

$$\cos^6\theta_\chi = \frac{1}{\chi}\cdot\frac{1}{6\pi^2\phi_{V,1}}\left(\frac{S_\chi}{\sigma_{LV,\chi}}\right)^3, \qquad \text{(S8).}$$

Justified in the main text and section S1 above, the ratio between spreading coefficient, S_χ, and surface tension, $\sigma_{LV,\chi}$ remains constant for mixtures at different concentration, i.e. $S_\chi/\sigma_{LV,\chi} = const$. Thus the model for apparent contact angle with respect to χ is

$$\cos^6\theta_\chi = \frac{1}{\chi}\cos^6\theta_1. \qquad \text{(S9)}$$

Note that the volume of a printed oil drop with a contact angle θ and a contact radius R can be approximated as $V = \pi R^3(2 - 3\cos\theta + \cos^3\theta)/\sin^3\theta$.

S3. Particle Size Distribution by Dynamic Light Scattering

The mean particle size distribution of crude-hexadecane mixture was averaged over five independent samples at $\chi = 0.01$, 0.05, 0.1, 0.3 and 0.5. The measurements are obtained using dynamic light scattering (DLS) method. Two peaks corresponding to particles at 4 nm and ~1μm are observed. The results show that crude does contain nano-scale and micron-scale particles, while Hexadecane does not.

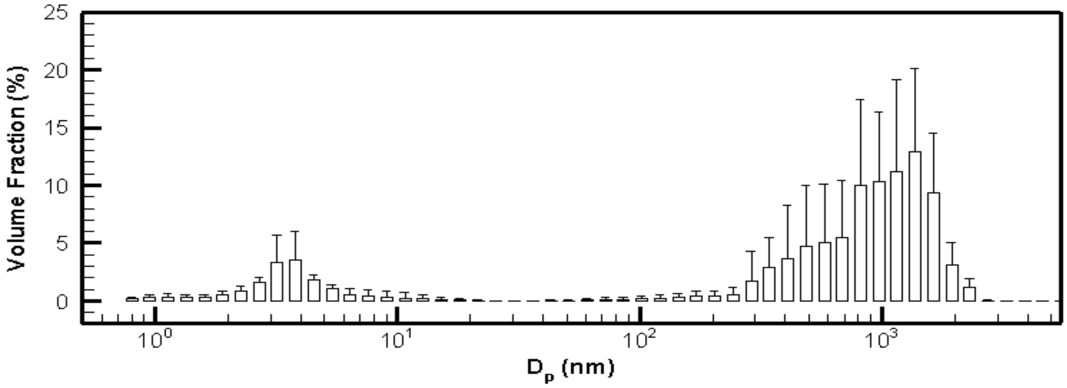

Fig. S2| Mean particle size distribution of crude oil measured by Dynamic Light Scattering (DLS). The mean distribution is averaged over measurements of samples at different concentration, χ, of crude in crude-hexadecane mixture. The samples are prepared at $\chi =$ 0.01, 0.05, 0.1, 0.3 and 0.5. The error bar is one standard deviation.

S4. Compute local curvatures of AFM measurement.

Due to the non-axisymmetric base of the printed drops like squares, conventional means of topology characterization using cross-sectional profiles are inadequate. Instead, we characterize the 2D surface, $z(x,y)$, directly by calculating the distribution of local principal radii of curvature, $R_1(x,y)$ and $R_2(x,y)$, or mean curvature, $H(x,y) = \frac{1}{2}\left(\frac{1}{R_1} + \frac{1}{R_2}\right)$, where x and y are the lateral positions. Recall that since both the Young-Laplace equation for a larger sessile drop and Kelvin

equation for micro/nano-colloidal particle contain the geometric term of H (or $1/r_{eq}$, where r_{eq} is the equivalent radius of curvature), the proposed quantification is justified.

To estimate the local H, we must first approximate the local principal radii, R_1 and R_2. We apply least square fit of a second order polynomial surface, $z(r_x, r_y; x, y) = k_{11}r_x^2 + k_{12}r_x r_y + k_{22}r_y^2 + k_1 r_x + k_2 r_y + k_0$ over a local surface centered around the position, (x, y), with an extent of (Δ_x, Δ_y), where r_x and r_y are distance originated from (x, y), i.e. $r_x \leq \Delta_x$ and $r_y \leq \Delta_y$. Note that the coefficients, k_{11}, k_{22} and k_{12}, represent the curvatures of a convex surface in the *x*, *y* and diagonal direction respectively. The principal curvature, κ_1 $(or\ 1/R_1)$ and κ_2 $(or\ 1/R_2)$, are then obtained by applying a coordinate transformation that rotates about z-axis allowing k_{12} to vanish. It yields the following relationship.

$$\kappa_1 = k_{11}\cos^2\theta - k_{12}\cos\theta\sin\theta + k_{22}\sin^2\theta$$
$$\kappa_2 = k_{11}\sin^2\theta + k_{12}\cos\theta\sin\theta + k_{22}\cos^2\theta \quad \text{(S10)}$$

where $\tan(2\theta) = (k_{22} - k_{11})/k_{12}^2$. The mean curvature is estimated as $H = (\kappa_1 + \kappa_2)/2$ or equivalently as $H = (k_{11} + k_{22})/2$. To ensure robust approximation and prevent spurious value of H from measurement uncertainties, coefficients of each polynomial surface are estimated over a sufficiently large window ($\Delta_x, \Delta_y \geq 2\mu m$ for all prints with different features).

Figure S3 elucidates the characterization procedure using the distribution of local mean radius of curvature, $r_{eq}(x, y) = \frac{1}{H}$ (or local mean curvature, H). The height profile of a fresh oil drop with a circular base (CIR50) is shown in Fig. S3a and the corresponding distribution of r_{eq} in Fig. S3b. The window for estimating r_{eq} is 3 μm in both directions. It is observed that the example drop has the apex height of 4.6 μm and a mean diameter of 56 μm, slightly larger than that of the feature in the stamp. Although the drop retains a general circular base (Fig. S3a), the elongation in AFM scanning direction (positive *x*) are clearly observed. In addition, striations caused by AFM probes can also be observed in r_{eq} (Fig. S3b) but less pronounced in profile (Fig. S3A). More than 80% of the center portion of the drop has constant mean radius of curvature, $r_{eq} = 182.8 \pm 8.9\ \mu m$, while along the periphery (~20%), r_{eq} increases rapidly above $400\ \mu m$ and transits to negative values. This trend is consistent regardless of the size and base shape (substantiated in §3.2) and suggests the oleophilic contact angle. Although due to the large measurement error near the periphery, the estimation of the contact angle and r_{eq} in this region is expected to be inaccurate, but it should provide us with qualitative information on drop shape. This trend can be further

elucidated with radial profile in Fig. S3c (red line), which is averaged over the azimuthal direction at the interval of 5° and superimposed with that of a best fit sphere. Note that the radial profile of drop agrees well within an error of $0.46\mu m$ or 0.5% with the profile of a sphere of an estimated radius, $R_{sp} = 96.7\mu m$. Recall that the r_{eq} of drop is $r_{eq} = \sim 1.89 R_{sp}$ in our measurement, while the r_{eq} of a sphere must maintain twice of R_{sp} in principle.

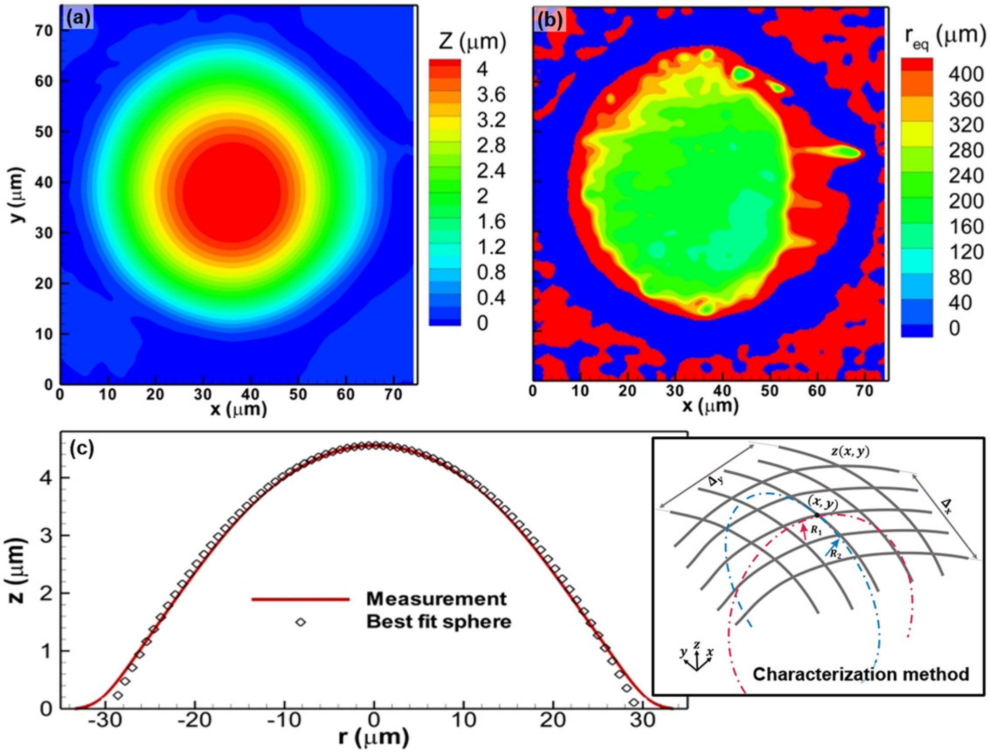

Fig. S3| Characterization of drop shape with mean radius of curvature, *H*. (a) surface profile of a $50\mu m$ crude oil drop, $z(x, y)$. (b) 2D distribution of mean radius of curvature, $r_{eq}(x, y)$, using local best fit of 2nd order polynomial convex surface. (c) Red line: Radial profile of oil drop averaged over the azimuthal direction, Symbol: Radial profile of the best-fit sphere. Inset: Schematics of method used for estimating mean radius of curvatures.

S5 Properties of printable oils

Printing material	Density, $\rho, (kg/m^3)$	Surface tension $\sigma, (mN/m)$	viscosity, μ, (cP)	Oh at 50um $\mu/(\rho\sigma d_f)^{1/2}$	Data Spec. Source
Fresh crude	856.2	26.1	10.1	0.30	Ref. [3]
10% Weathered crude	877	28	23.7	0.68	Ref. [3]
Hexadecane	770	27.5	3.4	0.10	Refs. [4,5]
80% glycerol water	1200	63.1	60.1	0.98	Refs. [6,7]
Olive oil	920	32	84	2.19	Refs. [7,8]
5W30	860	60	64	1.26	Refs [9,10]
10W40	865	99	94	1.44	Refs [9,10]
Vacuum oil	890	33	126	3.28	Refs [11]

Table S1| Material properties of selected oils in current experiment. Crude is Louisiana sweet used to emulate the Deep Horizon Spilled oil. The weathered crude is obtained by baking the fresh crude at 90C until 10% of initial mass is lost.

References

1 White, A. R. & Ward, T. Pattern search methods for pendant drops: Algorithms for rapid determination of surface tension and surfactant transport parameters. **485** 11 (2015).

2 Weon, B. M. & Je, J. H. Self-Pinning by Colloids Confined at a Contact Line. **110**, 028303 (2013).

3 Wang, Z. *et al.* Characteristics of spilled oils, fuels, and petroleum Products. (Environmental Protection Agency, North Carolina, 2003).

4 Hardy, R. C. Viscosity of n-Hexadecane **61**, 4 (1958).

5 Krämer, B. *et al.* Homogeneous nucleation rates of supercooled water measured in single levitated microdroplets **111**, 7 (1999).

6 Association, G. P. *Physical properties of glycerine and its solutions*. (Gulf Publishing Company, 1967).

7 Jones, A. Z. *Experimental surface tension values*, <http://physics.about.com/od/physicsexperiments/a/surfacetension_5.htm> (2015).

8 Bempah, A. & Hileman, E. J. Mean lifetime of an embryo in the homogeneous nucleation from solution of the tetracyanoplatinates(I1) of Barium, calcium and magnesium **51**, 9 (1973).

9 Du, W., Li, C., Nichols, K. P. & Ismagilov, R. F. Slip Chip. **9**, 7 (2009).

10 Ueda, E., Geyer, F. L., Nedashkivskaab, V. & Levkin, P. A. Droplet microarray: Facile formation of arrays of microdroplets and hydrogel micropads for cell screening applications. **12**, 7 (2012).

11 Griffing, E. M., Bankoff, S. G., Miksis, M. J. & Schluter, R. A. Electrohydrodynamics of thin flowing films. **128**, 8 (2005).
